# Dual Functioned Hexapeptide‐Coated Lipid‐Core Nanomicelles Suppress Toll‐Like Receptor‐Mediated Inflammatory Responses through Endotoxin Scavenging and Endosomal pH Modulation

**DOI:** 10.1002/advs.202301230

**Published:** 2023-04-20

**Authors:** Yuting Ji, Liya Sun, Yuan Liu, Yanhui Li, Tongxuan Li, Jiameng Gong, Xiali Liu, Huiqiang Ma, Jingying Wang, Bing Chen, Shan‐Yu Fung, Hong Yang

**Affiliations:** ^1^ The Province and Ministry Co‐Sponsored Collaborative Innovation Center for Medical Epigenetics Department of Pharmacology School of Basic Medical Sciences Intensive Care Unit of the Second Hospital Tianjin Medical University No. 22 Qixiangtai Road, Heping district Tianjin 300070 China; ^2^ Department of Immunology and Key Laboratory of Immune Microenvironment and Disease (Ministry of Education) School of Basic Medical Sciences Tianjin Medical University No. 22 Qixiangtai Road, Heping district Tianjin 300070 China; ^3^ Department of Pulmonary and Critical Care Medicine Shanghai General Hospital Shanghai Jiao Tong University School of Medicine No. 650 Xinsongjiang Road Shanghai 201620 China; ^4^ Intensive Care Unit of the Second Hospital Tianjin Medical University No. 22 Qixiangtai Road, Heping district Tianjin 300070 China

**Keywords:** acute lung injury, endotoxin scavenger, lipid nanomicelle, macrophage, nano‐therapy, Toll‐like receptor

## Abstract

Excessive activation of Toll‐like receptor (TLR) signaling pathways and the circulating endotoxin are key players in the pathogenesis of many acute and chronic inflammatory diseases. Regulation of TLR‐mediated inflammatory responses by bioactive nanodevices represents a promising strategy for treating these diseases. In searching for novel, clinically applicable nanodevices with potent TLR inhibitory activities, three types of hexapeptide‐modified nano‐hybrids with different cores of phospholipid nanomicelles, liposomes, and poly(lactic‐co‐glycolic acid) nanoparticles are constructed. Interestingly, only the peptide‐modified lipid–core nanomicelles (M‐P12) display potent TLR inhibitory activities. Further mechanistic studies disclose that lipid–core nanomicelles have a generic property to bind to and scavenge lipophilic TLR ligands including lipopolysaccharide to block the ligand–receptor interaction and down‐regulate the TLR signaling extracellularly. In addition, the peptide modification enables M‐P12 a unique capability to modulate endosomal acidification upon being endocytosed into macrophages, which subsequently regulates the endosomal TLR signal transduction. In an acute lung injury mouse model, intratracheal administration of M‐P12 can effectively target lung macrophages and reduce lung inflammation and injuries. This work defines a dual mechanism of action of the peptide‐modified lipid–core nanomicelles in regulating TLR signaling, and provides new strategies for the development of therapeutic nanodevices for treating inflammatory diseases.

## Graphical abstract

Regulation of Toll‐like receptor (TLR)‐mediated inflammatory responses by nanodevices represents a promising strategy for treating inflammatory diseases. Herein, a generic property of lipid–core nanomicelles in endotoxin scavenging and TLR signaling modulation is discovered. Furthermore, a dual functioned hexapeptide‐coated lipid–core nanomicelle is developed to potently suppress TLR‐mediated inflammatory responses in vitro and in vivo.

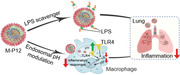

## 1 Introduction

The pattern recognition receptors (PRRs) are key players in the innate immune responses of the host defense mechanisms against the invasion of pathogens and foreign harmful substances to our body.^[^ ^1^ ^]^ They can detect a unique repertoire of distinct foreign molecules termed pathogen‐associated molecular patterns (PAMPs) to trigger defensive immune reaction.^[^ ^2^ ^]^ Toll‐like receptors (TLRs) are a family of PRRs, which can recognize and respond to a variety of PAMPs, such as the bacterial cell wall components lipopolysaccharide (LPS) and lipoproteins, and bacterial and viral RNA/DNA.^[^ ^3^ ^]^ In addition, TLRs can also respond to the damage‐associated molecular patterns (DAMPs) released from the injured host cells/tissues to mount inflammatory responses.^[^ ^4^ ^]^ Although TLR responsiveness is essential to control infection, excessive TLR activation often contributes to pathogenesis of many inflammatory diseases.^[^ ^5^ ^]^ For example, the excessive activation of TLR4 is associated with high morbidity of sepsis,^[^ ^6^ ^]^ and the removal of circulating LPS (TLR4 ligand) is therapeutic beneficial to septic patients.^[^ ^7^ ^]^ In addition, the LPS endotoxemia was found to be associated with an increased risk of cardiovascular diseases and obesity.^[^ ^8^ ^]^ Moreover, the serum LPS could participate in the pathogenesis of pulmonary embolism, pulmonary heart disease, and venous thromboembolism.^[^ ^9^ ^]^ Therefore, attenuating TLR signaling and/or antagonizing TLR ligands, particularly LPS, has emerged as a novel therapeutic strategy for treating various inflammatory diseases.

To modulate TLR signaling, small‐molecule inhibitors,^[^ ^10^ ^]^ oligonucleotides,^[^ ^11^ ^]^ lipid A analogs,^[^ ^12^ ^]^ antibodies and miRNA^[^ ^13^ ^]^ have been developed to either block the ligand–receptor interaction extracellularly or inhibit the intracellular TLR signaling transduction. However, none of these TLR inhibitors/antagonists are approved for clinical uses. This is probably because very often, multiple TLR pathways rather than a single TLR are involved in the pathogenic inflammation. In the case of circulating endotoxin (LPS)‐induced systematic inflammation and multi‐organ failure, removing endotoxin is expected to be a promising way to improve the survival of septic patients.^[^ ^14^ ^]^ As the progression to septic shock and multi‐organ failure occurs within 48 h with a high mortality rate, the removal of endotoxin should be timely and efficient. Based on these facts, to develop novel, potent, and multiaction TLR inhibitors that can both scavenge endotoxin and attenuate TLR signal transduction may provide an ideal solution to regulate TLR‐mediated pathogenic inflammation that still lacks effective clinical treatments.

Modulation of TLR‐mediated immune responses using nanodevices has become an attractive targeted strategy to treat inflammatory diseases. Compared with the conventional molecular drugs, nanodevices have preferred pharmacokinetics and biodistribution profiles, contributing to better targeting efficiency, higher therapeutic efficacy and less potential side effects. Taking the advantages of these characteristics, we previously developed a novel nano‐hybrid with potent inhibitory activity on TLR signaling.^[^ ^15^ ^]^ This nano‐hybrid (P12) is made of 13‐nm gold nanoparticles (GNPs) coated with hexapeptides. P12 is capable of inhibiting the activation of transcription factors nuclear factor *κ*B/activator protein 1 (NF‐*κ*B/AP‐1) and interferon regulatory factors (IRFs) in multiple TLR signaling pathways in macrophages, and down‐regulating the inflammatory responses.^[^ ^15^ ^]^ P12 also targets alveolar macrophages and exhibits protective effects on LPS‐induced lung inflammation and injury.^[^ ^16^ ^]^ However, the GNP core of P12 is not biodegradable, which represents a challenge for its clinical translation. To overcome this challenge, we endeavor to introduce different biodegradable cores to replace GNPs in P12 to facilitate the translation of this novel discovery to clinical uses. Moreover, we anticipate that the new cores may impart the nano‐hybrids with beneficial functionality.

In this study, we first constructed three different nano‐hybrids by replacing the GNP core of P12 with a biodegradable modality to facilitate the clinical translation while maintaining the TLR inhibitory activities. We chose lipid–core nanomicelles, liposomes (Lipo) and poly(lactic‐co‐glycolic acid) (PLGA) nanoparticles as the new cores, and conjugated them with the hexapeptide Pep12 to fabricate the new nano‐hybrids M‐P12, Lipo‐P12 and PLGA‐P12, respectively. To our surprise, the peptide‐coated lipid–core nanomicelles M‐P12 exhibited the most potent inhibitory activity on TLR signaling and TLR‐mediated proinflammatory cytokine production in macrophages among the three nano‐hybrids while the PLGA‐P12 had little effects on TLR inhibition. Further mechanistic studies using different lipid–core nanomicelles disclosed that the nanomicelles themselves (without Pep12 modification) could scavenge TLR ligands, playing a critical role in TLR inhibition. In addition, the peptide coating imparted the lipid–core nanomicelles with an endosomal pH modulatory activity, enabling these nanomicelles to attenuate the endosomal TLR signaling. Finally, the in vivo anti‐inflammatory effects and the macrophage targeting capability of M‐P12 were evaluated in a LPS‐induced acute lung injury (ALI) mouse model. This study discovered a new bioactivity of lipid–core nanomicelles as endotoxin scavenger in modulating TLR‐mediated inflammatory responses. It also identified M‐P12 as a novel class of dual functioned biodegradable nano‐modulator for TLR signaling, which may hold great promise for the treatment of various acute and chronic inflammatory diseases.

## 2 Results

### 2.1 Fabrication and Characterization of Pep12‐Modified Nanoparticles and their Regulatory Activity on TLR4 Signaling

**Figure 1:**
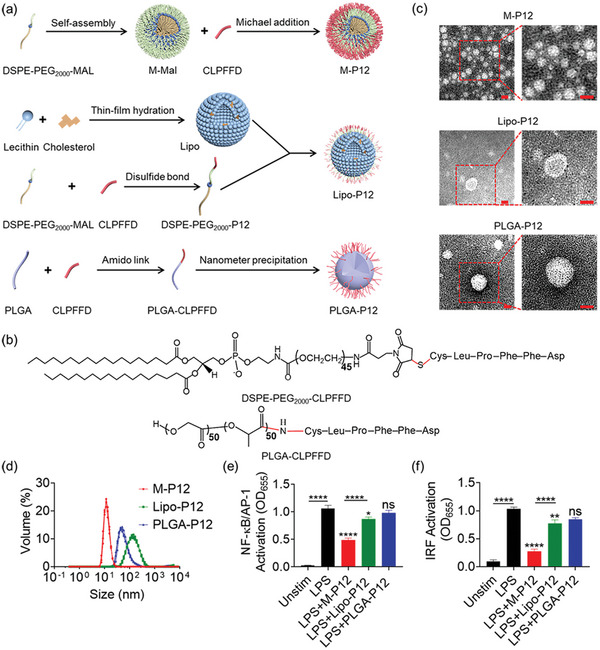
Fabrication and characterization of the hexapeptide‐modified nano‐hybrids M‐P12, Lipo‐P12 and PLGA‐P12 and their inhibitory activity on TLR4 signaling. a) A schematic diagram showing the fabrication of M‐P12, Lipo‐P12, and PLGA‐P12. b) The chemical structures of DSPE‐PEG_2000_ and PLGA conjugated with Pep12 (CLPFFD) through the Michael addition reaction and amide bond formation, respectively. c) The TEM images of M‐P12, Lipo‐P12, and PLGA‐P12 with a zoom‐in field on the right; scale bar = 20 nm. d) The hydrodynamic size distribution of the three nano‐hybrids by DLS measurements. The effects of M‐P12, Lipo‐P12 and PLGA‐P12 on the LPS‐induced activation of NF‐*κ*B/AP‐1 (e) and IRF (f) in the THP‐1 reporter cell‐derived macrophages; *N* = 3. LPS = 10 ng mL^−1^, all nano‐hybrids (phospholipids or polymers): 0.2 mg mL^−1^; ns: not significant, *p < 0.05, **p < 0.01, ****p < 0.0001 versus LPS unless otherwise specified.

We previously developed a new class of bioactive peptide‐GNP hybrids, P12, capable of inhibiting multiple TLR signaling pathways.^[^ ^15^ ^]^ However, the non‐biodegradable GNP core of P12 may hinder the clinical translation of P12. To overcome this problem, we constructed three versions of peptide (Pep12)‐modified nano‐hybrids by replacing the GNP core with different cores that were made of clinically applicable materials in this study (**Figure** **1**a): M‐P12, Lipo‐P12, and PLGA‐P12. We anticipated that these new cores may also bring additional functionality to the nano‐hybrids. M‐P12 was made of self‐assembled distearoyl‐phosphatidylethanolamine‐poly(ethylene glycol) (2000)‐maleimide (DSPE‐PEG_2000_‐MAL) nanomicelles (M‐MAL) that were conjugated with Pep12 (CLPFFD) on the surface by Michael addition reaction between the maleimide of the DSPE‐PEG_2000_‐MAL and the thiol group of the cysteine (C) residue at the N‐terminal of Pep12 (Figure 1b). The nuclear magnetic resonance hydrogen spectroscopy (^1^H NMR) was applied to confirm the conjugation of Pep12 on the nanomicelle surface. The disappearance of the maleimide peak (at 6.7 ppm) in DSPE‐PEG_2000_‐MAL and the appearance of the benzene peak of the peptide (at 7.1–7.2 ppm) in DSPE‐PEG_2000_‐Pep12 suggested the complete conjugation of Pep12 to the nanomicelle (Figure S1a, Supporting Information). Lipo‐P12 was fabricated by inserting DSPE‐PEG_2000_‐Pep12 into the phospholipid bilayer of the DSPE liposomes (Lipo). PLGA‐P12 was constructed by conjugating Pep12 to PLGA monomers via amide bond formation (Figure 1b), followed by nano‐precipitation to form peptide‐modified PLGA nanoparticles. The appearance of the benzene peak in the NMR spectra of PLGA‐P12 suggested the successful conjugation of Pep12 to PLGA (Figure S2a, Supporting Information).

All three nano‐hybrids had a spherical shape with a relatively uniform particle size distribution (Figure 1c). The hydrodynamic sizes of M‐P12, Lipo‐P12 and PLGA‐P12 measured by dynamic light scattering (DLS) were 15.8 ± 0.6, 113.8 ± 1.0 and 64.9 ± 1.2 nm, respectively (Figure 1d). The zeta potential analysis showed that M‐P12 and Lipo‐P12 had a more negative potential value than the unmodified M‐MAL and Lipo, respectively (Figure S1b,c, Supporting Information), while the zeta‐potential of PLGA‐P12 was less negative than that of the PLGA core (Figure S2b, Supporting Information); this again demonstrated the success of Pep12 conjugation to these cores. The critical micelle concentration (CMC) of M‐P12 was found to be 4.3 ± 0.6 µg mL^−1^, lower than that of DSPE‐PEG_2000_‐OCH_3_ (5.2 ± 1.9 µg mL^−1^) (Figure S1d,e, Supporting Information), suggesting that M‐P12 had relatively good stability against dilution.

Next, we assessed the inhibitory activity of these nano‐hybrids on the TLR4 signaling using the NF‐*κ*B/AP‐1 and IRF reporter THP‐1 cell‐derived macrophage systems. Unexpectedly, M‐P12 and Lipo‐P12 were able to inhibit the LPS‐induced activation of NF‐*κ*B/AP‐1 and IRF, while PLGA‐P12 was not (Figure 1e,f); it appeared that the inhibitory effect of M‐P12 was the strongest among the three. It is worth noting that these nano‐hybrids did not have any cytotoxicity at the experimental conditions (Figure S3, Supporting Information), and M‐P12 alone did not induce the activation of NF‐*κ*B/AP‐1 and IRF in macrophages (Figure S4, Supporting Information). These surprising but encouraging findings suggested that the lipid–core of M‐P12 may play an important role in TLR inhibition. Accordingly, M‐P12 was selected among the three for the rest of the experiments in this study.

### 2.2 The Potent Inhibitory Activity of M‐P12 on TLR4 Signaling and Cytokine Production in Macrophages

**Figure 2:**
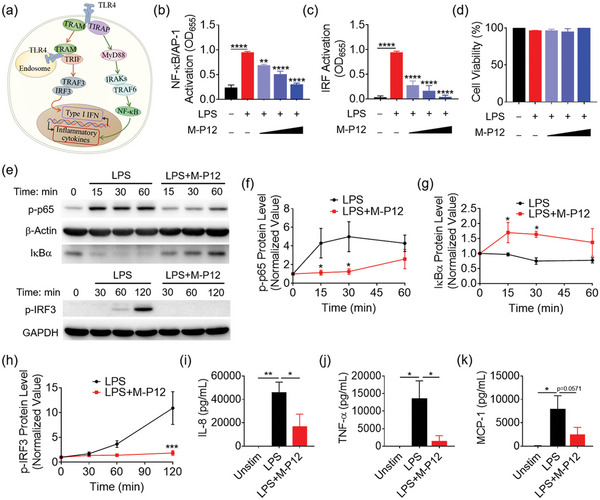
The inhibitory activity of M‐P12 on TLR4 signaling pathway in THP‐1 cell‐derived macrophages. a) A schematic diagram presenting the two arms of the TLR4 signaling pathway. Inhibition of the LPS‐induced NF‐*κ*B/AP‐1 activation (b) and IRF3 activation (c) in TLR4 signaling by M‐P12; M‐P12 (phospholipids): 0.05, 0.1 and 0.2 mg mL^−1^; *N* = 3. d) The effect of M‐P12 on the viability of the THP‐1 reporter cell‐derived macrophages upon LPS stimulation; *N* = 3. e) The inhibitory effects of M‐P12 on the phosphorylation of p65 (p‐p65) and degradation of I*κ*B*α* for NF‐*κ*B activation and the phosphorylation of IRF3 (p‐IRF3) for IRF activation over time (0–2 h) under LPS stimulation in THP‐1 cell‐derived macrophages via immunoblotting; *β*‐actin and GAPDH as the internal control. The time course densitometry analysis of p‐p65 (f), I*κ*B*α* (g), and p‐IRF3 (h) quantified from (e); *N* = 3. Inhibition of IL‐8 (i), TNF‐*α* (j), and MCP‐1 (k) production by M‐P12 upon LPS stimulation for 24 h in THP‐1 cell‐derived macrophages; *N* = 3 for IL‐8 and TNF‐*α*, *N* = 5 for MCP‐1. LPS = 10 ng mL^−1^, M‐P12 (phospholipids): 0.2 mg mL^−1^; ns = not significant, *p < 0.05, **p < 0.01, ***p < 0.001, ****p < 0.0001 versus LPS unless otherwise specified.

TLR4 signaling transduction consists of two pathways: the myeloid differentiation factor 88 (MyD88)‐dependent pathway and the MyD88‐independent (via Toll/interleukin‐1 receptor domain‐containing adaptor‐inducing interferon beta, TRIF) pathway (**Figure** **2**a).^[^ ^17^ ^]^ The former activates the key transcription factor NF‐*κ*B, which can be probed by the phosphorylation of the subunit p65 and the degradation of the inhibitor unit I*κ*B*α*; the latter requires the signals through endosomes to trigger IRF3 activation for the production of type I interferon (IFN).

Using the THP‐1 reporter cell‐derived macrophages, we confirmed that M‐P12 could inhibit LPS‐induced activation of NF‐*κ*B/AP‐1 and IRF in a concentration dependent manner (Figure 2b,c), but did not affect the cell viability at all (Figure 2d). The inhibitory activity of M‐P12 on TLR4 was further verified by immunoblotting (Figure 2e). The results showed that M‐P12 significantly down‐regulated the phosphorylation of p65 (p‐p65) (Figure 2e,f) and reduced I*κ*B*α* degradation (Figure 2e,g) under LPS stimulation for up to 60 min; the LPS‐induced phosphorylation of IRF3 (p‐IRF3) was completely inhibited by M‐P12 over a period of 120 min (Figure 2e,h). Furthermore, M‐P12 was able to decrease the production of various proinflammatory cytokines including interleukin‐8 (IL‐8), tumor necrosis factor (TNF)‐*α*, and monocyte chemoattractant protein (MCP)‐1 upon LPS stimulation for 24 h (Figure 2i–k). These results demonstrated that M‐P12 had potent inhibitory activity on TLR4 signaling and the associated inflammatory responses in macrophages.

### 2.3 M‐P12 Had a Broad TLR Inhibitory Activity Preferentially on the Endosomal TLR Pathways

**Figure 3:**
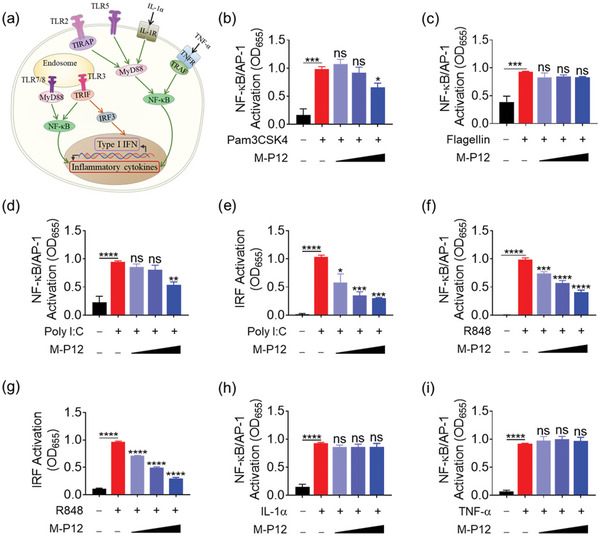
The preferential inhibitory activity of M‐P12 on TLR2 and endosomal TLR signaling pathways in THP‐1 reporter cell‐derived macrophages. a) The schematic diagram of TLR2, TLR5, and endosomal TLR signaling pathways as well as TNF‐α and IL‐1α‐mediated signaling pathways. The effects of M‐P12 on the activation of NF‐*κ*B/AP‐1 stimulated by Pam3CSK4 (10 ng mL^−1^) (b) and flagellin (1 µg mL^−1^) (c) in TLR2 and TLR5 signaling pathways, respectively. The inhibitory effects of M‐P12 on Poly I:C (50 µg mL^−1^)‐induced NF‐*κ*B/AP‐1 (d) and IRF (e) activation in TLR3 signaling pathway. The inhibitory effects of M‐P12 on R848 (10 µg mL^−1^)‐induced NF‐*κ*B/AP‐1 (f) and IRF (g) activation in TLR7/8 signaling pathway. ; M‐P12 had no effects on the NF‐*κ*B/AP‐1 activation induced by IL‐1*α* (100 ng mL^−1^) (h) or TNF‐*α* (20 ng mL^−1^) (i). *N* = 3; M‐P12 (phospholipids): 0.05, 0.1, and 0.2 mg mL^−1^; ns: not significant, *p < 0.05, **p < 0.01, ***p < 0.001, ****p < 0.0001 versus TLR agonists, IL‐1α or TNF‐α unless otherwise specified.

In addition to TLR4, we also wondered whether M‐P12 was capable of inhibiting other TLR pathways. Among the TLR family, TLR2 and TLR5 are expressed on the cell surface, while TLR3 and TLR7/8 are on the endosomal membranes; they signal through either MyD88‐dependent or MyD88‐independent pathways^[^ ^18^ ^]^ (**Figure** **3**a). Using different TLR agonists, we found that M‐P12 treatment at a concentration of 200 µg mL^−1^ could reduce Pam3CSK4 (TLR2 ligand)‐induced activation of NF‐*κ*B/AP‐1 (Figure 3b) but had no effects on the TLR5 signaling triggered by flagellin (Figure 3c). On the other hand, M‐P12 exhibited stronger inhibitory effects on both TLR3 and TLR7/8 signaling stimulated by Poly I:C and R848, respectively, in a concentration‐dependent fashion (Figure 3d–g). These results indicated that M‐P12 had a relatively broad TLR inhibitory spectrum preferentially on the endosomal TLR pathways.

To verify if such an inhibitory activity of M‐P12 was specific to TLRs, other important inflammatory signaling pathways were examined. The TNF receptor (TNFR) and the IL‐1 receptor (IL‐1R) can respond to TNF‐*α* and IL‐1*α*/*β*, respectively, from the cell surface to activate NF‐*κ*B.^[^ ^19^ ^]^ Interestingly, it was found that M‐P12 did not affect either TNF‐*α* or IL‐1*α* stimulated NF‐*κ*B activation (Figure 3h,i). These results suggested that M‐P12 primarily affected the lipid‐containing ligands (i.e., LPS and Pam3CSK4) triggered‐TLR signaling as well as the endosomal TLR pathways, but had no effects on other surface receptors IL‐1R and TNFR.

### 2.4 The TLR Inhibitory Activity of M‐P12 Was Primarily Determined by the Lipid‐Core Nanomicelles

**Figure 4:**
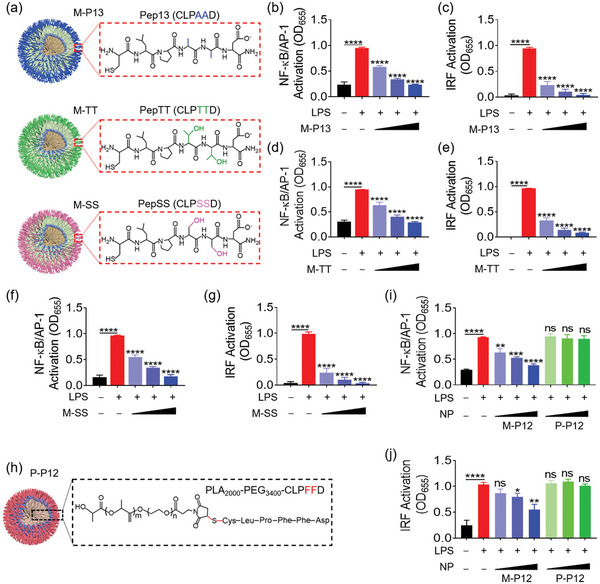
The inhibitory effects of lipid–core nanomicelles conjugated with different peptides and peptide‐modified polymeric nanomicelles on TLR4 signaling pathway in THP‐1 reporter cell‐derived macrophages. a) A schematic diagram showing lipid‐based nanomicelles modified with other peptides Pep13, PepTT and PepSS to form M‐P13, M‐TT and M‐SS, respectively. b–g) The inhibitory effects of M‐P13 (b,c), M‐TT (d,e), and M‐SS (f,g) on LPS‐induced NF‐*κ*B/AP‐1 (b,d,f) and IRF (c,e,g) activation. h) A schematic diagram of Pep12‐modified PLA_2000_‐PEG_3400_ polymeric nanomicelles P‐P12. P‐P12 had no effects on inhibition of LPS‐induced NF‐*κ*B/AP‐1 (i) and IRF (j) activation in comparison with M‐P12. *N* = 3; LPS = 10 ng mL^−1^, all nanomicelles (phospholipids or polymers): 0.05, 0.1, and 0.2 mg mL^−1^; ns: not significant, *p < 0.05, **p < 0.01, ***p < 0.001, ****p < 0.0001 versus LPS unless otherwise specified.

Our previous study have shown that the bioactivity of P12 can be programmed by the amino acid sequence of Pep12 on the GNP surface.^[^ ^15^ ^]^ When Pep12 was replaced by other peptides, such as Pep13 (CLPAAD), PepTT (CLPTTD) and PepSS (CLPSSD), the peptide‐GNP hybrids did not exhibit inhibitory activity on TLR signaling transduction. To examine if this phenomenon is still held for M‐P12, we replaced Pep12 with Pep13, PepTT and PepSS to fabricate M‐P13, M‐TT and M‐SS nanomicelles, respectively (**Figure** **4**a); the physicochemical characterization of these nanomicelles was shown in Figure S5, Supporting Information, and their inhibitory activity on TLR4 signaling was assessed on the THP‐1 reporter cell‐derived macrophages. To our surprise, M‐P13, M‐TT and M‐SS all significantly decreased LPS‐induced activation of NF‐*κ*B/AP‐1 and IRF (Figure 4b–g). In addition, they were able to inhibit TLR2 (Figure S6, Supporting Information), TLR3 and TLR7/8 signaling pathways (Figures S7–S9, Supporting Information) as well. This suggested that the observed TLR inhibitory activity of M‐P12 was not mainly determined by the conjugated peptides on the surface, and thus we speculated that the lipid core of the nanomicelles might be the key contributor.

To test our speculation, we employed an amphiphilic polymer PLA_2000_‐PEG_3400_ to form polymeric micelles of which surface was modified with Pep12, named P‐P12 (Figure 4h). Compared with M‐P12 made of the phospholipid micellar core, P‐P12 consisting of a polymeric micellar core had no inhibitory effects on TLR4‐mediated NF‐*κ*B/AP‐1 and IRF activation (Figure 4i,j); nor could P‐P12 suppress TLR2, TLR3 and TLR7/8 signaling pathways (Figure S10, Supporting Information). These results concurred with our speculation that the phospholipid core rather than the surface peptides of M‐P12 was the main contributing factor for the TLR inhibitory activity. This also supported the findings that PLGA‐P12 did not inhibit TLR4 signaling as shown in Figure 1.

**Figure 5:**
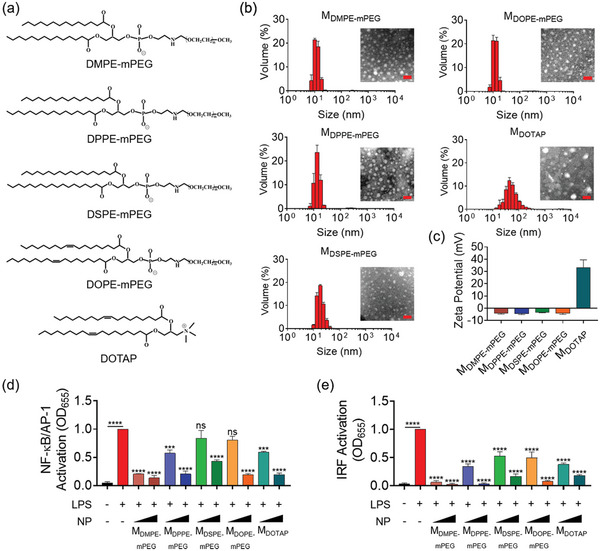
The inhibitory effects of different lipid–core nanomicelles without peptide modification on TLR4 signaling. a) The chemical structures of five different lipid monomers: the negatively charged DMPE‐mPEG, DPPE‐mPEG, DSPE‐mPEG and DOPE‐mPEG, and the positively charged DOTAP, which self‐assemble into the nanomicelles *M*_DMPE‐mPEG_, *M*_DPPE‐mPEG_, *M*_DSPE‐mPEG_, *M*_DOPE‐mPEG_ and *M*_DOTAP_, respectively. b) The hydrodynamic size distribution and TEM images (inset) of the five nanomicelles; scale bar = 100 nm. c) The zeta‐potential of the five nanomicelles. The inhibitory effects of these nanomicelles on the LPS‐induced activation of NF‐*κ*B/AP‐1 (d) and IRF (e) in the THP‐1 reporter cell‐derived macrophages. *N* = 3; LPS = 10 ng mL^−1^, all nanomicelles (phospholipids): 0.05 and 0.2 mg mL^−1^; ns: not significant, ***p < 0.01, ****p < 0.0001 versus LPS unless otherwise specified.

Since the phospholipid core was essential for the inhibitory ability of M‐P12, we were inquisitive about whether this activity was specific to the chemical property of the lipid core. For such a purpose, five nanomicelles were constructed using different amphiphilic lipid molecules: dimyristoyl‐phosphatidylethanolamine‐mPEG (DMPE‐mPEG), dipalmitoyl‐phosphatidylethanolamine‐mPEG (DPPE‐mPEG), DSPE‐mPEG, dioleoyl‐phosphatidylethanolamine‐mPEG (DOPE‐mPEG), and dioleoyl‐trimethylammonium propane (DOTAP) (**Figure** **5**a). These lipid amphiphiles were different in the carbon chain length (13–17 carbons), the presence of unsaturated bonds in the carbon chain or the PEGylated modification and ionic charges in the hydrophilic group. The DLS analysis showed that these lipid–core nanomicelles, *M*_DMPE‐mPEG_, *M*_DPPE‐mPEG_, *M*_DSPE‐mPEG_, *M*_DOPE‐mPEG_ and *M*_DOTAP_, had a relative uniform particle size of 12.1 ± 0.2, 14.1 ± 1.1, 19.4 ± 1.2, 12.3 ± 0.7 and 108.2 ± 4.7 nm, respectively, and they all displayed a spherical morphology under transmission electron microscopy imaging (Figure 5b). Note that the relatively wide size distribution of *M*_DOTAP_ was probably because the unPEGylated DOTAP amphiphiles tended to form some large vesicles that skewed the DLS measurements. Except for *M*_DOTAP_ that had positive charges on the surface due to the ammonium group, the zeta potential of all other nanomicelles was weakly negative for their negatively charged phosphoryl groups (Figure 5c). Very interestingly, all these nanomicelles were able to inhibit LPS‐induced activation of NF‐*κ*B/AP‐1 and IRF (Figure 5d,e); it was found that the negatively charged nanomicelles also had inhibitory activity on TLR2 (Figure S11a, Supporting Information), TLR3 (Figure S11b,c, Supporting Information) and TLR7/8 (Figure S11d,e, Supporting Information), but not on TLR5 (Figure S11f, Supporting Information). These results confirmed that the lipid–core nanomicelles were capable of inhibiting TLR signaling, and such a capability did not depend on the surface modification or the chemical properties of the lipid cores.

As the *M*_DMPE‐mPEG_ exhibited the highest potency in attenuating TLR4 signaling among the tested unmodified lipid–core nanomicelles, we chose it as the unmodified control nanomicelle in the following studies.

### 2.5 The Mechanisms of Action of M‐P12 in Inhibiting TLR Signaling

**Figure 6:**
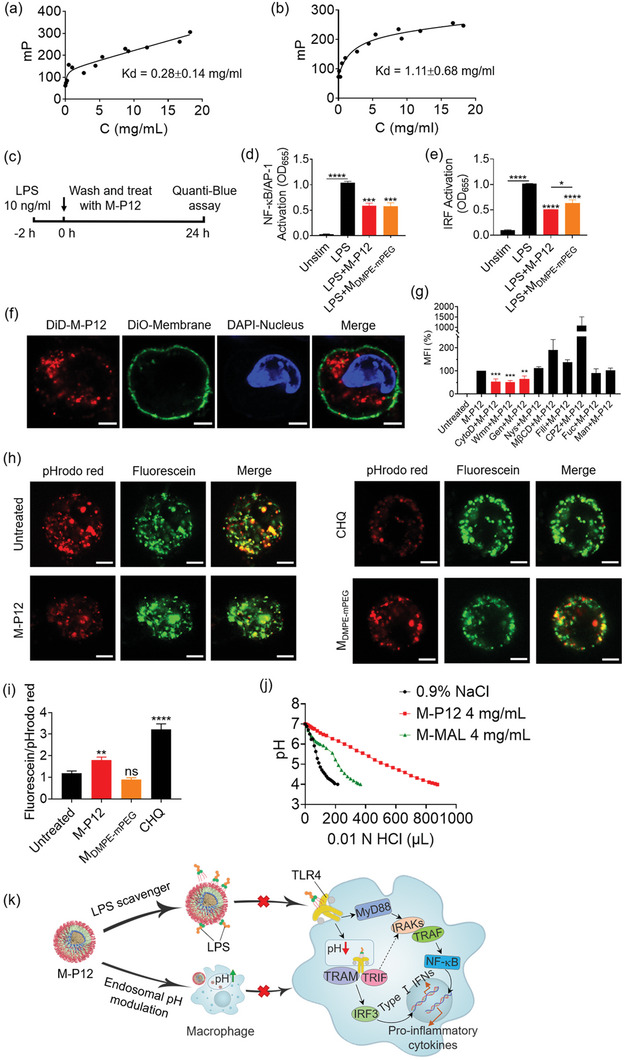
The extracellular and intracellular mechanisms of action of M‐P12 on the inhibition of TLR signaling in the THP‐1 cell‐derived macrophages. The fluorescence polarization profiles of FITC‐labelled LPS (10 ng mL^−1^) mixed with various concentrations (phospholipids) of M‐P12 (a) and *M*_DMPE‐mPEG_ (b); the dissociation constant Kd values were obtained from non‐linear fitting of the curves. c) The experimental procedure of LPS (10 ng mL^−1^) stimulation for 2 h followed by LPS removal and nanomicelle treatment of M‐P12 and *M*_DMPE‐mPEG_ for 24 h in THP‐1 reporter cell‐derived macrophages. The inhibition of NF‐*κ*B/AP‐1 (d) and IRF (e) activation by post‐treatments of M‐P12 and *M*_DMPE‐mPEG_ 2 h after LPS stimulation shown in (c); all nanomicelles (phospholipids): 0.2 mg mL^−1^, LPS = 10 ng mL^−1^. f) Confocal images of THP‐1 cell‐derived macrophages treated with DiD (red)‐labelled M‐P12 (DiD‐M‐P12); the cell membranes and the nucleus were stained with DiO (green) and DAPI (blue), respectively; DiD‐M‐P12 (phospholipids): 0.2 mg mL^−1^; scale bar = 5 µm. g) The flow cytometry analysis of DiD‐M‐P12 uptake (mean fluorescence intensity of DiD) in the macrophages with/without various endocytotic pathway inhibitors for 3 h; Cyto D: cytochalasin D (3 µm), Wmn: wortmannin (10 µm), Gen: genistein (10 µm), Nys: nystatin (10 µm), M*β*CD: methyl‐*β*‐cyclodextrin (5 mm), Fili: filipin (10 µm), CPZ: chlorpromazine (10 µm), Fuc: fucoidan (25 µg mL^−1^), Man: mannose (500 µg mL^−1^); DiD‐M‐P12 (phospholipids): 0.2 mg mL^−1^; *N* = 4–5. h) Confocal images of THP‐1 cell‐derived macrophages treated with M‐P12, *M*_DMPE‐mPEG_ and chloroquine (CHQ, 30 µm) for 6 h; the endosomal pH was probed by pHrodo red (red) (10 µg mL^−1^) and fluorescein (green) (20 µg mL^−1^) labelled dextran; all nanomicelles (phospholipids): 0.2 mg mL^−1^; scale bar = 5 µm. i) The quantification of the green‐to‐red ratio of the fluorescence signals; *N* = 75–120 cells. j) The pH titration of M‐P12 and M‐MAL in normal saline (0.9% NaCl); M‐P12 and M‐MAL (phospholipids): 4 mg mL^−1^. k) A scheme summarizing the dual mechanism of action of TLR4 inhibition by M‐P12 through scavenging LPS and modulating endosomal acidification in macrophages. *N* = 3 unless otherwise specified, *p < 0.05, **p < 0.01, ***p < 0.001, ****p < 0.0001 versus LPS or M‐P12 or untreated control unless otherwise specified.

So far, we have shown the potent inhibitory activity of M‐P12 and the lipid–core nanomicelles on TLR signaling. This inhibitory activity was primarily dependent on the lipid–core, and had preference toward TLR2, TLR4 and endosomal TLR3 and TLR7/8. It has been reported that TLR signaling transduction can be stopped by either blocking the interaction between the receptor and the corresponding ligand or interfering with the intracellular signaling cascades.^[^ ^20^ ^]^ Based on the above findings, we first hypothesized that the lipid–core of M‐P12 may have high affinity to lipid‐based TLR ligands (e.g., LPS), serving as a scavenger to block the ligand–receptor interaction. To test this hypothesis, the polarization of fluorescein isothiocyanate (FITC)‐labelled LPS was measured upon the introduction of M‐P12 at various concentrations. As shown in **Figure** **6**a, the FITC polarization values (mP) increased and reached a plateau with the increasing concentration of M‐P12, indicating the binding of LPS to M‐P12. It is worth noting that the observed binding of LPS to M‐P12 was not due to the labelled FITC, as M‐P12 was able to inhibit the FITC‐LPS‐induced activation of NF‐*κ*B/AP‐1 and IRF in a concentration dependent manner (Figure S12, Supporting Information). A similar FITC polarization profile was also observed for the *M*_DMPE‐mPEG_ (without Pep12 modification), indicating that LPS could bind to *M*_DMPE‐mPEG_ as well (Figure 6b). The nonlinear fitting of the two polarization profiles revealed the equilibrium dissociation constant Kd of M‐P12 and *M*_DMPE‐mPEG_ as 0.28 ± 0.14 and 1.11 ± 0.68 mg mL^−1^, respectively, suggesting that the affinity of LPS to M‐P12 was stronger than that to *M*_DMPE‐mPEG_. Similarly, using the FITC‐labelled Poly I:C (TLR3 ligand), we found that Poly I:C could also weakly bind to M‐P12 with a Kd value of 2.42 ± 1.57 mg mL^−1^ (Figure S13, Supporting Information). These results suggested that M‐P12 and *M*_DMPE‐mPEG_ could interact and scavenge lipophilic TLR ligands to block the TLR stimulation and hence down‐regulate TLR signaling.

In addition to scavenging TLR ligands for TLR inhibition, we also wondered if M‐P12 and *M*_DMPE‐mPEG_ could modulate the intracellular signal transduction during TLR activation. To answer this question, a post‐treatment approach was conducted, where the THP‐1 reporter cell‐derived macrophages were stimulated with LPS for 2 h, followed by LPS removal and nanomicelle treatment for 24 h (Figure 6c). It was found that both M‐P12 and *M*_DMPE‐mPEG_ still inhibited the LPS‐induced activation of NF‐*κ*B/AP‐1 and IRF in the absence of LPS (Figure 6d,e). Interestingly, M‐P12 exhibited a stronger inhibitory effect for IRF activation than *M*_DMPE‐mPEG_. Through the same post‐treatment approach, M‐P12 also reduced the intracellular TLR3 signal transduction (Figure S14, Supporting Information). These results indicated that the nanomicelles were capable of modulating the intracellular TLR signaling transduction in addition to the ability of sequestering TLR ligands.

In order to interfere with the TLR signal transduction intracellularly, these nanomicelles must be present inside the cells. In fact, it was clearly seen by the confocal microscopy that the DiD probe labelled M‐P12 was internalized by macrophages (Figure 6f), and the uptake of M‐P12 was energy‐dependent (Figure S15, Supporting Information), indicating that the uptake of M‐P12 was an active process (e.g., endocytosis) by the cells. Using different inhibitors to block the internalization pathways, we found that the uptake of M‐P12 was significantly reduced by the macropinocytosis inhibitors wortmannin (Wmn) and cytochalasin D (CytoD), and the caveolae/lipid raft‐mediated endocytosis inhibitor genistein (Gen) (Figure 6g), suggesting that the macropinocytosis and caveolae/lipid raft‐mediated endocytosis were involved in the M‐P12 uptake by macrophages.

Because we previously found that the peptide‐GNP hybrid P12 could modulate the endosomal pH to inhibit TLR activation, we rationalized that M‐P12 may have a similar mechanism as well. The change in the endosomal pH can be measured by two pH‐sensitive fluorescence probes (pHrodo red‐ and fluorescein‐labeled dextrans) upon M‐P12 and *M*_DMPE‐mPEG_ treatment. The increase in the ratio of the fluorescence intensity of fluorescein to pHrodo red indicates the elevation of endosomal pH.^[^ ^21^ ^]^ Chloroquine (CHQ) that is capable of preventing endosomal acidification served as the positive control. As shown in Figure 6h, the red color (pHrodo red) became dimmer in the CHQ and M‐P12 treated groups when compared with the untreated cells, but remained unchanged with *M*_DMPE‐mPEG_ treatment; the ratio of green‐to‐red fluorescence increased in both CHQ and M‐P12 treated cells compared with the untreated group (Figure 6i), indicating an increase in endosomal pH. This endosomal pH modulation was not observed in cells treated with *M*_DMPE‐mPEG_ (Figure 6h,i) nor with M‐P13, M‐TT and M‐SS (Figure S16, Supporting Information), indicating that such an effect was specific to the conjugated peptides (i.e., Pep12) on the nanomicelles. A pH titration test was also performed to confirm that M‐P12 did have a higher pH‐buffering capacity than the unmodified lipid–core M‐MAL (Figure 6j). Based on the above results, we proposed a working model of TLR inhibition in macrophages by M‐P12 through a dual mechanism: i) TLR ligand scavenging to stop ligand–receptor interaction extracellularly; and ii) endosomal pH modulation to inhibit intracellular signaling (Figure 6k).

### 2.6 M‐P12 Inhibited Lung Inflammation by Targeting Alveolar Macrophages in the LPS‐Induced ALI Mouse Model

**Figure 7:**
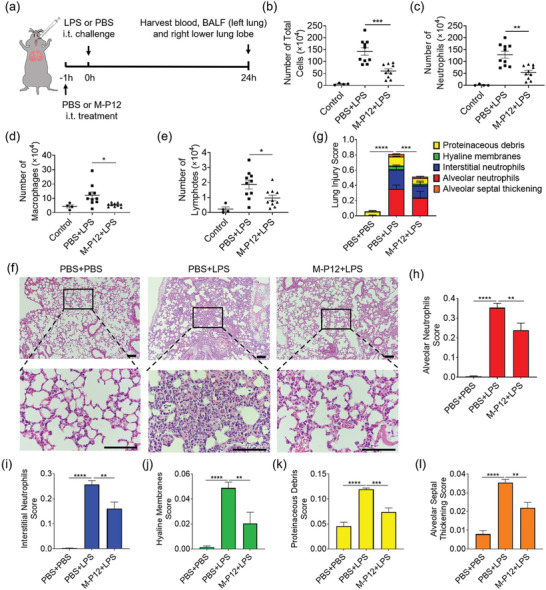
M‐P12 reduced the lung inflammation and injury in LPS‐induced ALI mice. a) The scheme of the LPS‐induced ALI mouse model; M‐P12 treatment was given intratracheally 1 h before LPS challenge, and the BALF and lung were analyzed 24 h later. The analysis of the number of total cells (b), neutrophils (c), macrophages (d), and lymphocytes (e) in the BALF. f) The histological images of lung sections stained with H & E; scale bar = 100 µm. The overall lung injury score (g) obtained from (f) based on the 5 pathophysiological characteristics: the alveolar neutrophils (h), interstitial neutrophils (i), hyaline membranes (j), proteinaceous debris (k), and alveolar septal thickening (l); *N* = 5 per group. LPS = 10 mg kg^−1^, M‐P12 (phospholipids): 0.5 mg kg^−1^; *p < 0.05, **p < 0.01, ***p < 0.001, ****p < 0.0001.

It has been found that TLR activation in macrophages plays a critical role in the pathogenesis of ALI/acute respiratory distress syndrome (ARDS).^[^ ^22^ ^]^ Since M‐P12 showed potent activity on TLR inhibition in vitro, we anticipated that M‐P12 may have protective effects on LPS triggered lung inflammation and injury in vivo. Using a classical LPS‐induced ALI mouse model (**Figure** **7**a), we found that M‐P12 significantly reduced the number of total inflammatory cells, neutrophils, macrophages, and lymphocytes in the bronchoalveolar lavage fluid (BALF) under LPS challenge (Figure 7b–e). The histopathological analysis on the peribronchiolar and perivascular inflammatory cells infiltration revealed the decrease in the degree of lung injury by M‐P12 (Figure 7f), which was quantified by the damage score of five histological characteristics (Figure 7g), including alveolar neutrophils, interstitial neutrophils, hyaline membranes, protein debris and alveolar septal thickening (Figure 7h–l). These results indicated that M‐P12 was effective in controlling acute lung inflammation. Similar protective effects were also found for the *M*_DMPE‐mPEG_ treatment on the same ALI mouse model (Figure S17, Supporting Information). It should be noted that although both M‐P12 and *M*_DMPE‐mPEG_ exhibited potent inhibitory effects on TLR signaling in vitro and protected mice from LPS‐induced lung inflammation and injury, M‐P12 was relatively more stable than the unmodified nanomicelles in terms of their changes in zeta potential and hydrodynamic sizes over time (Figure S18, Supporting Information).

**Figure 8:**
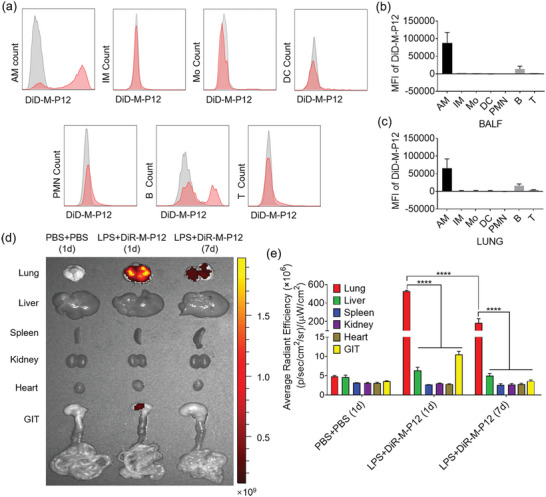
The macrophage targeting capability and biodistribution of M‐P12 in ALI mice after intratracheal administration. a) The DiD fluorescence histograms of DiD‐M‐P12 in various immune cells analyzed by flow cytometry: AM (alveolar macrophage), IM (interstitial macrophage), Mo (monocyte), DC (dendritic cell), PMN (polymorphonuclear cell), B (B cell), and T (T cell); the DiD‐M‐P12 treated group shown in red and the untreated group shown in grey. The quantitative analysis of the mean fluorescence intensity of DiD‐M‐P12 in various immune cells in the BALF (b) and lung tissues (c); DiD‐M‐P12 (phospholipid): 0.5 mg kg^−1^, DiD: 1.96 µg kg^−1^; *N* = 6. d) Ex vivo fluorescence images of major organs/tissues of the ALI mice treated with DiR‐labelled M‐P12 (DiR‐M‐P12) for 1 day and 7 days; healthy mice treated with an equal volume of PBS for 1 day as control; the DiR fluorescence signals (dark red to yellow color) were overlayed on the bright field images. e) Quantification of DiR fluorescence intensity in different organs in each treatment group from (d); the fluorescence signals were acquired at 800 nm with the excitation of 745 nm and were measured in radiance counts per centimeter square per second per steradian (p/s/cm^2^/sr); *N* = 3; DiR‐M‐P12 (phospholipids): 2.5 mg kg^−1^, DiR: 15.2 µg kg^−1^; ****p < 0.0001.

Next, we conducted the multi‐color flow cytometry analysis to identify different cell types in the BALF and lung of ALI mice (Figure S19, Supporting Information), and analyzed the uptake of the DiD‐labelled M‐P12 in these cells (**Figure** **8**a). Quantitative analysis showed that M‐P12 was preferentially taken up by alveolar macrophages in the BALF and lung; the uptake was much less in the interstitial macrophages, monocytes, dendritic cells, neutrophils, B cells and T cells (Figure 8b,c). This indicated that M‐P12 could primarily target alveolar macrophages after intratracheal administration to the lung.

To investigate the biodistribution of M‐P12 in vivo, M‐P12 was labelled with the DiR fluorescence dye (DiR‐M‐P12), and administered intratracheally to the lung; the major organs/tissues were collected for ex vivo imaging on Day 1 and Day 7 after LPS challenge. It was clearly seen that M‐P12 was mainly accumulated in the lung with some trace found in the gastrointestinal tract on Day 1, while the fluorescence signals were only seen in the lung 7 days after administration (Figure 8d). The fluorescence intensity quantification analysis showed the same results, where the fluorescence intensity of DiR was significantly higher in the lung than in other organs/tissues even after a week (Figure 8e). However, it should be noted that the DiR fluorescence intensity in the lung dropped significantly (>50%) from Day 1 to Day 7, indicating that the majority of administered M‐P12 could be excreted out of the body in a week. These results suggested that M‐P12 could target alveolar macrophages to elicit the anti‐inflammatory activity with a preferential distribution in the lung, and then excreted out of the body.

In addition to the ALI mouse model, we also found that M‐P12 was capable of reducing the intestinal inflammation in a mouse model of ulcerative colitis (Figure S20, Supporting Information), indicating the robust therapeutic potential of M‐P12 in different inflammatory diseases.

## 3 Discussion

TLRs can recognize PAMPs (e.g., bacterial LPS, flagellin, and microbial or viral RNA/DNA) and DAMPs to launch inflammatory reactions.^[^ ^3^, ^4^ ^]^ The overwhelming uncontrolled TLR‐mediated inflammatory responses have been found to contribute to the pathogenesis of many inflammatory disorders including ALI/ARDS, inflammatory bowel disease (IBD), atherosclerosis, and asthma.^[^ ^20^ ^]^ However, there are no TLR inhibitors or antagonists that have been approved for clinical uses. Herein, we discovered that lipid–core nanomicelles were capable of attenuating multiple TLR signaling pathways by scavenging the TLR ligands to block the ligand–receptor interaction for signal transduction. The modification of these nanomicelles with a special hexapeptide could introduce an additional mechanism of action on preventing endosomal acidification. This peptide‐modified nanomicelles M‐P12 showed effective targeting capability to lung macrophages and reduced lung inflammation and injury in a LPS‐induced ALI mouse model, representing a potent anti‐inflammatory nano‐hybrid.

### 3.1 The Generic Anti‐Inflammatory Activity of Lipid‐Core Nanomicelles through a Novel Mechanism of Scavenging TLR Ligands

Nanomicelles are self‐assembled nanostructures of amphiphilic molecules often consisting of a hydrophobic carbon chain linked with a hydrophilic group. Lipid–core nanomicelles are usually made of self‐assembled PEGylated phospholipids such as DSPE‐PEG. DSPE‐PEG nanomicelles are found to be promising drug carriers owing to their small size, biodegradability, desired pharmacokinetics and thermodynamic stability, and good biocompatibility.^[^ ^23^ ^]^ In fact, the DSPE‐PEG_2000_ has been approved for clinical use in three doxorubicin liposomal formulations (Doxil, LipoDox, and Thermodox) in the US and Europe.^[^ ^24^ ^]^ Based on these advantages, nanodevices made of DSPE‐PEG have been applied to carry therapeutic peptides for the treatment of inflammatory and autoimmune diseases.^[^ ^25^ ^]^ For example, DSPE‐PEG_2000_ micelles were conjugated with the human glucagon‐like peptide‐1 to treat ALI and IBD;^[^ ^26^ ^]^ they could also be loaded with an anti‐inflammatory vasoactive intestinal peptide as a potential treatment for rheumatoid arthritis.^[^ ^27^ ^]^ Despite the promising therapeutic potential of these nanomicelles as nanodrug carriers, their innate immunomodulatory activities have been merely explored.

In this study, we discovered that the lipid–core nanomicelles (without carrying any therapeutics) had generic anti‐inflammatory activities due to the novel capability of scavenging TLR ligands (Figures 5d,e and 6b; Figure S11, Supporting Information). All these nanomicelles (*M*_DMPE‐mPEG_, *M*_DPPE‐mPEG_, *M*_DSPE‐mPEG_, *M*_DOPE‐mPEG_, and *M*_DOTAP_) could reduce the activation of NF‐*κ*B and IRFs triggered by different TLR ligands (LPS, PAM3CSK4, Poly I:C, and R848) regardless of the length and the saturated status of the aliphatic side chains or PEGylation and ionic charges of the hydrophilic groups (Figure 5; Figure S11, Supporting Information). Interestingly, the protein‐based TLR5 ligand, flagellin, could not be sequestered by these nanomicelles (Figure S11f, Supporting Information), suggesting that such a scavenging activity may be ligand specific according to the ligand lipophilicity (i.e., aliphatic and amphiphilic structure). Furthermore, the addition of different hexapeptides on the surface of these lipid–core nanomicelles did not affect their ability to sequester TLR ligands and to attenuate TLR signaling pathways (Figures 2, 3, and 4a–g; Figures S6–S9, Supporting Information). It should be noted, however, that the polymeric nanomicelles (P‐P12) formed by PLA‐PEG‐Pep12 and the polymeric nanoparticles PLGA‐P12 had no effects on inhibiting TLR4 signaling (Figures 1e,f and 4h–j), indicating that the lipid cores were essential for these nanomicelles to sequester lipophilic TLR ligands. In fact, such an activity has been observed in liposomal nanoparticles,^[^ ^28^ ^]^ supporting our findings that the lipid–core nano‐assemblies were capable of scavenging endotoxin to inhibit TLR signaling.

Our previous studies have shown that the peptides coated on the GNPs determined the TLR inhibitory activity of peptide‐GNP nano‐hybrids. The hydrophobic amino acids (e.g., phenylalanine, F) in the peptide sequence enabled the inhibitory activity of the GNPs, whereas less hydrophobic (e.g., alanine, A) or the hydrophilic ones (e.g., threonine, T; serine, S) did not.^[^^15b]^ To our surprise, the peptide conjugated‐nanomicelles M‐P13, M‐TT and M‐SS all displayed similar TLR4 inhibitory activity (Figure 4a–g), which suggested that the endotoxin scavenging ability of the lipid core was probably robust enough to overrun the effects of the surface modification by hexapeptides.

### 3.2 The Dual Mechanism of M‐P12 on Inhibiting TLR Signaling

TLR signaling can be regulated at two levels. One is at the extracellular level to either antagonize the TLR ligands or directly interact with the receptors to block the ligand–receptor signals. It can also be intervened at the intracellular level, where the signal transduction is stopped by blocking the protein–protein interaction or the key protein activity to down‐regulate the signaling cascades.

To inhibit the TLR signaling extracellularly, various anti‐endotoxin therapies have been developed to antagonize LPS or directly block its interaction with the receptor. Even before the discovery of TLR4, human antiserum was found to sequester LPS and substantially reduce deaths from gram‐negative bacterial infections in 1982.^[^ ^29^ ^]^ Encouraged by this finding, several anti‐endotoxin therapies, including antibodies,^[^ ^30^ ^]^ phospholipid emulsion,^[^ ^31^ ^]^ the bactericidal/permeability‐increasing protein (BPI)^[^ ^32^ ^]^ and chemical compounds were applied to block the toxic effects of lipid A. The closest to success (in a phase III clinical trial) was Eritoran, a synthetic analogue of the lipid A portion of LPS, which was developed to bind TLR4 and inhibit its activation by LPS.^[^ ^33^ ^]^ Unfortunately, all these attempts have failed in clinical trials on septic patients.

On the other hand, small molecule inhibitors were developed to intervene with the TLR signal transduction intracellularly. For example, TAK‐242 (Resatorvid) was discovered to block the interaction between TLR4 and the adaptor proteins, the Toll‐interleukin‐1 receptor (TIR) domain containing adaptor protein (TIRAP) and TRIF‐related adaptor molecule (TRAM), to seize the signal transduction.^[^ ^33^ ^]^ However, it failed the clinical trials in patients with severe sepsis, although the drug was well‐tolerated.^[^ ^34^ ^]^ MicroRNAs were identified to efficiently regulate TLR signaling pathways,^[^ ^35^ ^]^ but precisely delivering them to the target cells was still a big challenge for their clinical uses. The antimalarial drugs, hydroxychloroquine sulfate and chloroquine, were found to inhibit the endosomal TLR signaling (TLR7/8/9) by modulating the endosomal acidification,^[^ ^36^ ^]^ but their efficacy in treating sepsis or virus‐induced ALI/ARDS has not been clearly defined yet.

Different from the conventional TLR inhibitors/antagonists, the developed peptide‐coated lipid–core nanomicelles (M‐P12) in this work displayed a dual mechanism of action on inhibition of TLR signaling. While the lipid core of M‐P12 could scavenge the TLR ligands extracellularly, the conjugated Pep12 (CLPFFD) enabled the capability of M‐P12 on endosomal pH modulation to attenuate intracellular TLR signaling pathways (Figure 6). We previously found that the surface modification of Pep12 on GNPs could promote the cellular uptake of GNPs in macrophages; the internalization of enormous Pep12‐modified GNPs (P12) could neutralize the protons in the endosomes and increase the endosomal pH.^[^ ^15^ ^]^ The blockade of endosomal acidification in turn inhibited the endosomal TLR signaling (e.g., TLRs 3, 4, and 7/8) because the acidic environment in endosomes is required for the recruitment of adaptor molecules to trigger signal transduction.^[^ ^38^ ^]^ Similar to P12, M‐P12 was found to accumulate in the endosomes and block the endosomal acidification (Figure 6f–i). However, the DMPE‐PEG nanomicelles without Pep12 modification did not affect the endosomal pH (Figure 6h,i). These findings suggested that the negatively charged aspartate (D) of Pep12 was critical to the neutralization of protons in endosomes, resulting in the blockade of endosomal acidification. Note that this activity was Pep12 specific as DSPE‐PEG nanomicelles modified with other hexapeptides (i.e., M‐P13, M‐TT, and M‐SS) could not modulate endosomal pH either (Figure S16, Supporting Information).

For the bacterial infection caused inflammatory diseases, the invading bacteria can be well‐controlled by a variety of anti‐microbial agents in the clinics, but the bacteria‐associated endotoxin as well as the excessive inflammatory immune responses are often the harmful factors exacerbating the situation. With a dual mechanism of action, it is expected that the developed M‐P12 can sequester the endotoxin while reducing the inflammatory responses, which is promising to treat inflammatory complications in many infectious conditions.

### 3.3 The Peptide‐Modified Lipid‐Core Nanomicelles as Promising Agents for Treating Various Acute and Chronic Inflammatory Diseases

Since M‐P12 exhibits a broad spectrum of anti‐inflammatory activity with a novel dual mechanism, it holds great potential for treating various acute and chronic inflammatory diseases. It has been found that excessive activation of TLRs and the circulating endotoxin are key players in the pathogenesis of many acute and chronic inflammatory diseases such as sepsis, ALI/ARDS, and IBD.^[^ ^38^ ^]^ Specifically, the overactivation of TLR2, TLR3, TLR4, and TLR9 by PAMPs has been reported to participate in the infection‐associated ARDS.^[^ ^38^, ^39^ ^]^ The circulating LPS is the key factor contributing to sepsis.^[^ ^7^ ^]^ The pathogenic bacteria, particularly *Escherichia coli*, in the gut can drive intestinal inflammation in IBD.^[^ ^40^ ^]^ Therefore, antagonizing LPS and reducing innate inflammatory responses are predicted to be a promising strategy to control the harmful inflammation in these critical pathogenic conditions.

We anticipated that the peptide‐conjugated lipid–core nanomicelles, M‐P12, could be a beneficial anti‐inflammatory therapy with the following advantages. M‐P12 is made of self‐assembled DSPE‐PEG in the core, which is approved by FDA for clinical uses in the preparation of liposomes, micelles and functional nanoparticles.^[^ ^42^ ^]^ The lipid–core nanomicelles can effectively scavenge lipophilic TLR ligands. The conjugation of Pep12 to the lipid–core nanomicelles can enhance the micelle stability (Figure S18, Supporting Information) and enable the ability to modulate endosomal pH (Figure 6h,i). Furthermore, the peptide modification does not impair the endotoxin scavenging capability of the nanomicelles. More importantly, the intratracheally administrated M‐P12 can target lung macrophages, reduce lung inflammation, and protect lungs from acute injuries in a LPS‐induced ALI mouse model (Figures 7 and 8). Interestingly, a very recent study demonstrated that the conjugation of Pep12 to a fluoroalkyl chain allowed the formation of fluorous‐tag peptide nano‐assemblies that exhibited anti‐oxidant and anti‐inflammatory activities.^[^ ^43^ ^]^ Taken together, this work discovered the inhibitory activity of lipid–core nanomicelles on TLR signaling pathways, which contributes to the fundamental understanding of the lipid‐based nanodevices in regulating immune responses. It also provided a novel class of anti‐inflammatory therapeutic nano‐hybrids to treat various acute and chronic inflammatory diseases.

## 4 Conclusions

In this study, we successfully replaced the gold core of the previously developed peptide‐GNP hybrid P12 with a biodegradable phospholipid micellar core, and demonstrated the TLR inhibitory activity of the new hybrid M‐P12 in vitro and in vivo. We first showed that the Pep12‐modified nanomicelles (M‐P12) exhibited stronger TLR inhibitory activity in macrophages than other nano‐hybrids with a liposomal core (Lipo‐P12) or a polymeric core (PLGA‐P12). Moreover, the inhibitory activity of M‐P12 did not depend on the amino acid sequences of the modifying peptides on the surface, rather on the characteristics of the nanomicellar cores. Further mechanistic studies revealed that M‐P12 had a dual mechanism of action through binding to and scavenging TLR ligands (e.g., LPS and Poly I:C) to block the extracellular ligand–receptor signaling, while regulating the intracellular endosomal acidification, leading to down‐regulation of TLR signal transduction. Finally, we demonstrated the macrophage targeting capability, preferred lung distribution, and the anti‐inflammatory effects of M‐P12 in the LPS‐induced ALI mouse model. This study embraced our understanding of lipid‐based bioactive nano‐hybrids in immune regulation, and provided new strategies for the treatment of acute and chronic inflammatory diseases.

## 5 Experimental Section

### Materials

DSPE‐PEG_2000_‐MAL and DSPE‐PEG_2000_‐OCH3 were purchased from AVT (Shanghai, China). Soya lecithin was purchased from Shanghai Taiwei Pharmaceutical Co., Ltd (Shanghai, China). Cholesterol was purchased from Sangon Biotech (Beijing, China). Other phospholipids, including DMPE‐mPEG, DPPE‐mPEG, DOPE‐mPEG and DOTAP, were obtained from Peng Sheng Biological (Shanghai, China). PLA_2000_‐PEG_3400_‐MAL and FITC‐labelled LPS were purchased from Xi'an ruixi Biological Technology Co., Ltd (Xi'an, China). PLGA was obtained from Jinan Daigang Biomaterial Co., Ltd (Jinan, China). All peptides were synthesized from Nanjing Jietai Biological Co., Ltd (Nanjing, China). *N*‐hydroxysulfosuccinimide sodium salt (NHS) and 1‐ethyl‐3‐(3‐dimethylaminopropyl)‐carbodiimide hydrochloride (EDC) were purchased from Meilunbio (Dalian, China). RPMI 1640 medium, phosphate buffered saline (PBS) and fetal bovine serum (FBS) were purchased from Biological Industries (Kibbutz Beit Haemek, Israel). L‐glutamine and sodium pyruvate were from Gibco (Grand Island, NY, USA). The human monocytic THP‐1 cell line was obtained from ATCC (Rockefeller, MD, USA). THP‐1 reporter cell lines (XBlue and ISG), LPS, fluorescein‐labelled LPS, Poly I:C (high molecular weight, HMW), fluorescein‐labelled Poly I:C, resiquimod (R848), Pam3CSK4, phorbol 12‐myristate 13‐acetate (PMA), Zeocin, and QUANTI‐Blue solution were from InvivoGen (San Diego, CA, USA). Recombinant human TNF‐*α* and IL‐1*α* were obtained from Peprotech (Rocky Hill, NJ, USA). MTS assay was purchased from Promega (Madison, WI, USA). The primary antibodies against phosphorylated p65 (p‐p65, 3033S), I*κ*B*α* (9242S), p‐IRF3 (4947S), *β*‐actin (8457S), and GAPDH (2118S) as well as the HRP conjugated anti‐rabbit (7074S) antibodies were purchased from Cell Signaling Technology (Boston, MA, USA). The RIPA lysis buffer, Halt protease and phosphatase inhibitor cocktail, the Coomassie Plus (Pierce) and pHrodo red and fluorescein‐labelled dextran (10 000 MW) were from Thermo Fisher Scientific (Waltham, MA, USA). Human ELISA kits of IL‐8, TNF‐*α* and MCP‐1 were purchased from eBioscience (San Diego, CA, USA). Chloroquine (CHQ), wortmannin (Wmn), fucoidan (Fuc), mannan (Man), genistein (Gen), methyl‐*β*‐cyclodextrin (M*β*CD) and chlorpromazine (CPZ) were purchased from Sigma (Sant‐Louis, MO, USA). Cytochalasin D (CytoD) and filipin III (Fili) were purchased from Cayman (Ann Arbor, MI, USA), while nystatin (Nys) was from MCE (Monmouth, NJ, USA). 3,3″‐dioctadecyloxacarbocyanine perchlorate (DiO) and 2‐(4‐amidinophenyl)‐6‐indolecarbamidine dihydrochloride (DAPI) were purchased from Beyotime (Shanghai, China). 1,1″‐dioctadecyl‐3,3,3″,3″‐tetramethylindodicarbocyanine perchlorate (DiD), Liu stain and red blood cell (RBC) lysis buffer were purchased from Solarbio Science & Technology (Beijing, China). 1,1″‐dioctadecy‐l‐3,3,3″,3′‐tetramethylindotricarbocyaine iodide (DiR) was purchased from Biolebo Science & Technology (Beijing, China). Live‐Dead (L34962) was purchased from InvitroGen (Carlsbad, CA, USA). Fluorescence probe labelled antibodies against CD45 (563410), Gr1 (561084), CD11c (562782), CD19 (564509) and CD3 (563024) were purchased from BD (New York, NJ, USA), while fluorochrome conjugated anti‐CD11b (101215) and anti‐F4/80 (123110) antibodies were acquired from BioLegend (San Diego, CA, USA).

### Preparation and Characterization of Different Types of Nano‐Hybrids

Nanomicelles were prepared by the thin‐film hydration method. Briefly, the PEGylated lipid‐based monomers (DMPE‐PEG_2000_, DPPE‐PEG_2000_, DSPE‐PEG_2000_, DOPE‐PEG_2000_ and DOTAP) (10 mg) were dissolved in chloroform (2 mL) and dried at low pressure, 45 °C to form a film. It was then rehydrated with PBS (3 mL) at 55 °C followed by bath ultrasonication (Scientz, Ningbo, China) at 120 W for 5 min at room temperature to form nanomicelles: *M*_DMPE‐PEG_. *M*_DPPE‐PEG_, *M*_DSPE‐PEG_, *M*_DOPE‐PEG_, and *M*_DOTAP_.

For M‐P12 preparation, Pep12 (CLPFFD) solution (2 mL and 2 mg mL^−1^ in PBS) was added to the nanomicelles at a molar ratio of 1.5:1 (Pep12:DSPE‐PEG_2000_‐MAL). The conjugation between the sulfhydryl group of Pep12 and the maleimide group of DSPE‐PEG_2000_‐MAL was done by Michael addition reaction in the dark for 24 h at room temperature with gentle stirring. The resulting mixtures were dialyzed against PBS (pH 7.4) for 24 h to remove the unconjugated Pep12. The same procedure was applied to fabricate other peptide‐modified lipid–core nanomicelles M‐P13, M‐TT and M‐SS by replacing the peptide Pep12 with Pep13, PepTT and PepSS, respectively. For constructing P‐P12, the micellar core made of DSPE‐PEG_2000_‐MAL was substituted with the polymeric core made of PLA_2000_‐PEG_3400_‐MAL. For the preparation of DiD‐labelled M‐12, DiD (38.4 µg) was added during the thin‐film hydration method to fabricate nanomicelles. These DiD‐loaded nanomicelles were modified with Pep12, followed by dialysis (<3500 Da) for 24 h to remove excess DiD prior to further experiments. All nanomicelles were sterilized by filtration (0.22 µm, Millipore, Billerica, MA, USA) prior to all cellular and in vivo experiments.

Lipo‐P12 was prepared using the thin‐film hydration method. Soya lecithin (180 mg) and cholesterol (60 mg) were dissolved in chloroform, and the solvent was completely removed to obtain the thin‐film. It was rehydrated in PBS (10 mL, pH 7.4), and the solution was sonicated by a probe ultrasonicator (Scientz, Ningbo, China) at 150 W for 5 min at room temperature to obtain liposomes (Lipo). The unmodified liposomes were incubated with DSPE‐PEG_2000_‐Pep12 for 2 h at 60 °C to obtain Lipo‐P12. All liposomes were sterilized by filtration (0.22 µm, Millipore, Billerica, MA, USA), centrifugated at 14 000 rpm for 2 h and stored at 4 °C prior to use.

The polymeric nano‐hybrid PLGA‐P12 was prepared in the following procedures. *N*‐hydroxysuccinimide (NHS, 192 mg) and 1‐ethyl‐3‐(3‐dimethylaminopropyl) carbodiimide hydrochloride (EDC, 57 mg) were dissolved in dimethylformamide (DMF, 1 mL); the mixture was then added to PLGA‐DMF solution (12.5 mg mL^−1^, 4 mL) to allow for reaction for 15 min at 4 °C. Subsequently, Pep12 (8 mg) dissolved in DMF (1 mL) was added to react with PLGA monomers for 48 h at room temperature for Pep12 conjugation to PLGA. The PLGA and PLGA‐P12 nanoparticles were obtained by nano‐precipitation in acetone, where PLGA or PLGA‐P12 monomers (10 mg mL^−1^) in acetone were diluted (1:9) into ultrapure water, followed by acetone volatilized under stirring (500 rpm) for 3 h. The solution was centrifuged and freeze‐dried to obtain PLGA and PLGA‐P12 nanoparticles.

These nanodevices were imaged by a transmission electron microscope (HT7700, Hitachi, Tokyo, Japan) with an accelerating voltage of 80 kV to visualize their morphology. The hydrodynamic diameter and zeta potential were determined on a Zetasizer instrument (Nano ZS, Malvern, Worcestershire, UK). The conjugation of Pep12 to DSPE‐PEG_2000_‐MAL and PLGA was confirmed by nuclear magnetic resonance hydrogen spectroscopy (^1^H NMR) (AVANCE III, BRUKER, Karlsruhe, Germany).

### Critical Micelle Concentration (CMC) Measurement

The CMC of micelles was determined by analyzing the change in pyrene fluorescence. Aliquot of pyrene (Aladdin, Shanghai, China) in acetone (5 µg mL^−1^) was transferred into brown volumetric flasks and air‐dried at room temperature to remove the acetone. The micelles solution with monomer concentrations ranging from 0.1 to 2000 µg mL^−1^ were added into each flask with a final pyrene concentration of 0.5 µg mL^−1^. The emission fluorescence spectra (350–600 nm) of pyrene in different micelle solutions were collected using a fluorescence spectrophotometer (F‐7000, Hitachi, Tokyo, Japan) with an excitation wavelength of 334 nm. The pyrene fluorescence intensity ratios of the characteristic first peak (373 nm) to the third peak (384 nm) were plotted as a function of monomer concentration, where the CMC was determined at the cross section of two straight lines fitted to the profile.

### Cell Culture

THP‐1 cells were maintained in RPMI‐1640 medium supplemented with sodium pyruvate (1 mm), L‐glutamine (2 mm), and 10% FBS. The THP‐1‐XBlue and THP‐1‐ISG reporter cells were cultured with the complete culture medium supplemented with Zeocin (200 and 100 µg mL^−1^, respectively) every other passage to maintain the selection pressure. All cells were cultured in an incubator at 37 °C with 5% CO_2_. These cells were seeded into a 12‐well, 24‐well, or 96‐well culture plate and differentiated into macrophages with the presence of PMA (50 ng mL^−1^) for 24 h; they were then washed twice with PBS and rested for 48 h prior to further experiments.

### Reporter Cell Assay for the NF‐*κ*B/AP‐1 and IRF Activation Analysis

Reporter cells (1 × 10^5^ cells/well) were seeded in a 96‐well plate and differentiated into macrophages. They were treated with different TLR ligands, IL‐1*α* or TNF‐*α* in the presence or absence of various nanomicelles and nano‐hybrids for 24 h; the culture medium was collected (20 µL) and mixed with the QUANTI‐Blue solution (180 µL) in a 96‐well plate, which was incubated at 37 °C until the color changed from pink to dark blue. The absorption at 655 nm was measured using a microplate reader (TECAN, Mannedorf, Zurich, Switzerland). The color change indicated the activation of NF‐*κ*B/AP‐1 or IRF in comparison with the untreated control.

### Immunoblotting Analysis

THP‐1 cells (1 × 10^6^ cells/well) were seeded into a 12‐well plate and differentiated into macrophages. They were stimulated with LPS (10 ng mL^−1^) with or without M‐P12 (phospholipids: 0.2 mg mL^−1^) treatment for different time periods. The cells were then lysed with the Halt protease and phosphatase inhibitor cocktail supplemented RIPA buffer under ice‐cold condition. The total protein concentration was quantified using the Coomassie Plus Bradford assay and adjusted to the same level. The proteins were separated by 10% SDS‐PAGE and transferred to PVDF membranes. The membranes were blocked with 5% BSA in the TBS buffer containing 0.1% Tween 20 for 1 h at room temperature, followed by blotting with primary antibodies against *β*‐actin, I*κ*B*α*, phosphorylated p65 (p‐p65), GAPDH and p‐IRF3 at 4 °C overnight. They were then washed and blotted with HRP labelled secondary antibody for 1 h at room temperature, and the protein bands were imaged using the chemiluminescence method (ECL, Millipore, Billerica, MA, USA) on a ChemiDoc MP imaging system (Bio‐Rad, Hercules, CA, USA). The protein band densitometry was analyzed using ImageJ software (NIH, Bethesda, MD, USA).

### Cytokine Analysis

THP‐1 cells were seeded (5 × 10^5^ cells/well) and differentiated into macrophages in a 24‐well plate. After different treatments of TLR ligands with/without nanomicelles for 24 h, the culture medium was centrifuged (14 000 rpm, 4 °C, 30 min), collected and stored at −80 °C. The levels of IL‐8, TNF‐*α*, and MCP‐1 were quantified using ELISA kits according to the manufacturer's instructions.

### Fluorescence Polarization Experiments

To study the interaction of TLR ligands with the nanomicelles, FITC‐labelled LPS (1 µg mL^−1^) and fluorescein‐labeled Poly I:C (5 µg mL^−1^) were prepared in Tris buffer (1 m, pH 9.0), which were mixed with different concentrations of M‐P12 and *M*_DMPE‐mPEG_ (phospholipids: up to 18.2 mg mL^−1^) with a volume ratio of 1:99 in a black 96‐well flat plate and incubated at room temperature for 30 min. The fluorescence polarization value (mP) was measured using a microplate reader (TECAN, Mannedorf, Zurich, Switzerland) with an excitation wavelength at 485 nm and an emission signal acquired at 530 nm.

### Laser Confocal Fluorescence Microscopy

THP‐1 cells (2.4 × 10^5^ cells) were seeded in a 20 mm glass bottom dish (NEST, Wuxi, China) and differentiated into macrophages as previously described. To examine the cellular uptake of M‐P12, macrophages were treated with DiD‐labelled M‐P12 (0.2 mg mL^−1^ phospholipid and 0.77 µg mL^−1^ DiD) overnight, and stained with DiO for 20 min and DAPI for 5 min to label the cell membrane and nucleus, respectively. The fluorescence images were acquired on a confocal microscope (LSM900, Leica, Wetzlar, Hessen, Germany).

To assess the endosomal acidification, macrophages were incubated with pHrodo red (10 µg mL^−1^) and fluorescein‐labelled dextran (20 µg mL^−1^) in the presence of nanomicelles (phospholipid: 0.2 mg mL^−1^) or chloroquine (CHQ, 30 µm) treatment for 6 h. Cells were then washed 3 times with PBS and imaged on a confocal microscope (LSM900, Leica, Wetzlar, Hessen, Germany). The fluorescence intensities of pHrodo red (ex: 565 nm; em: 585 nm) and fluorescein (ex: 488 nm; em: 525 nm) were quantified by Image J software. For each condition, at least 60 cells from 3 independent experiments were quantified to obtain the intensity ratio of fluorescein to pHrodo red.

### Acid‐Base Titration of the Nanomicelles

The buffering effects of M‐P12 and the unmodified M‐MAL were determined by a standard acid–base titration method using a benchtop pH meter (PB‐10, Sartorius, Goettingen, Germany). M‐P12 and M‐MAL nanomicelles were re‐suspended in 0.9% NaCl solution at a concentration of 4 mg mL^−1^, with 0.9% NaCl solution as the control. All solutions (5 mL) were first adjusted to pH 7.0 with 0.1 n NaOH, and then titrated with 0.01 n HCl gradually until pH 4.0 was reached. The titration profile was obtained by plotting each recorded pH value with the total amount of HCl added.

### Cellular Uptake Pathway Analysis

To analyze the internalization pathways of M‐P12, THP‐1 cell‐derived macrophages were pretreated with various inhibitors 30 min prior to the addition of DiD‐labelled M‐P12 (phospholipids: 0.2 mg mL^−1^) for 3 h. These inhibitors included chlorpromazine (CPZ, 10 µm) for clathrin‐mediated endocytosis, nystatin (Nys, 10 µm), genistein (Gen, 200 µm), methyl‐*β*‐cyclodextrin (M*β*CD, 5 mm) and filipin III (Filipin, 10 µm) for caveolae/lipid raft‐mediated endocytosis, cytochalasin D (CytoD, 3 µm) and wortmannin (Wmn, 10 µm) for macropinocytosis, fucoidan (Fuc, 25 µg mL^−1^) for scavenger receptor‐mediated endocytosis and mannan (Man, 500 µg mL^−1^) for mannose receptor‐mediated endocytosis. After treatments, cells were washed and resuspended in PBS for flow cytometry analysis (BD FACSVerse, San Diego, CA, USA).

### LPS‐Induced ALI Murine Model

Male C57BL/6 mice (6–8 weeks) were purchased from SPF Biotechnology Co., Ltd (Beijing, China). All mouse studies were performed according to the guidelines of the Institutional Animal Care and Use Committee of Tianjin Medical University (TMUaMEC2020004). All procedures were conducted under 1% sodium pentobarbital anesthesia to minimize mouse suffering. Mice were randomly divided into three groups: PBS+PBS group, PBS+LPS group, and M‐P12+LPS group. After being anesthetized, the trachea of mice was exposed through a median neck incision, and M‐P12 (phospholipid: 0.5 mg kg^−1^) or an equal volume of PBS was injected into the trachea 1 h before LPS (10 mg kg^−1^, Sigma‐Aldrich, Sant‐Louis, MO, USA) challenge through the same route. Mice were sacrificed 24 h after the LPS challenge to harvest the BALF, blood serum and lung tissue to examine cell infiltration, cytokine production and lung injury severity.

### BALF Collection and Differential Cell Counting

The BALF collection was performed based on the following procedures. The chest cavity of mice was opened to collect blood from the heart apex. The right hilum was then ligated, and ice‐cold PBS (0.4 mL) was injected into the left lung lobe through the trachea twice; the BALF was collected and centrifuged at 1000 rpm for 10 min at 4 °C. The cell pellets were processed with red blood cell (RBC) lysis buffer and resuspended in PBS for cell counting analysis. The total BAL cells were counted on a hemocytometer; aliquots (50 µL) of cell suspension were centrifuged on a glass slide using a cytospin (Thermo Fisher Scientific, Waltham, MA, USA) and processed with Liu stain. The slides were imaged on an optical microscope (Nikon, Tokyo, Japan) for differential cell counting. A total of at least 300 cells were counted for each sample.

### Flow Cytometry Analysis of M‐P12 Uptake in Different Immune Cells in the Lung

DiD‐labeled M‐P12 (0.5 mg kg^−1^ phospholipid and 1.92 µg kg^−1^ DiD) was intratracheally injected into ALI mice. After 24 h, the BALF and the lung single cell suspension were collected. These samples were then stained with viability dye and fluorochrome‐labelled antibodies, including anti‐CD45, anti‐CD11b, anti‐F4/80, anti‐CD11c, anti‐Gr1, anti‐CD19 and anti‐CD3 for flow cytometry analysis (Fortessa, BD, San Diego, CA, USA).

### Lung Histology and Injury Score

The right lower lung lobe of mice was collected, fixed in 4% paraformaldehyde, dehydrated, and embedded in paraffin for sectioning. The tissue sections were stained with hematoxylin and eosin (H&E) and imaged on an optical microscope (Nikon, Tokyo, Japan). For each sample, at least 20 images at 400× amplification were blindly scored by three independent researchers based on five histopathological features: alveolar neutrophils, interstitial neutrophils, hyaline membranes, proteinaceous debris, and alveolar septal thickening.

### Biodistribution of M‐P12 in ALI Mice

To visualize M‐P12 in different organs, mice were intratracheal administered with DiR‐labelled M‐P12 (2.5 mg kg^−1^ phospholipid and 15.2 µg kg^−1^ DiR) or equal volume of PBS for 1 h prior to LPS (10 mg kg^−1^) or equal volume of PBS injection. After LPS challenge for 1 day and 7 days, mice were sacrificed and major organs/tissues including lung, liver, spleen, kidney, heart and gastrointestinal tract (G.I.T.) were harvested. The fluorescence imaging (excitation: 745 nm; emission: 800 nm) was performed on an IVIS SPECTRUM (Perkin Elmer, Hopkinton, MA, USA), and the fluorescence signals were overlayed on the bright field images.

### Statistical Analysis

The statistical analysis was performed using GraphPad Prism 7 by the student *t*‐test (for the comparison between two groups), nonlinear regression (curve fit), or one‐way ANOVA with Bonferroni post‐test whenever applicable. All the data were presented as means ± standard error of mean (SEM) with *N* ≥ 3. *p* value < 0.05 was considered statistically significant.

## Conflict of Interest

The authors declare no conflict of interest.

## Supporting information

Supporting Information

## Data Availability

The data that support the findings of this study are available from the corresponding author upon reasonable request.
