# Supplementary material for: Dual Functioned Hexapeptide‐Coated Lipid‐Core Nanomicelles Suppress Toll‐Like Receptor‐Mediated Inflammatory Responses through Endotoxin Scavenging and Endosomal pH Modulation
Source: Adv Sci (Weinh). 2023 Apr 20;10(19):2301230. doi: 10.1002/advs.202301230 (PMC10323664; doi:10.1002/advs.202301230)
Supplement: Supplementary file 1 — Supporting Information [file ADVS-10-2301230-s001.pdf]

ADVANCED SCIENCE
Open Access

Supporting Information

for *Adv. Sci.*, DOI 10.1002/advs.202301230

Dual Functioned Hexapeptide-Coated Lipid-Core Nanomicelles Suppress Toll-Like Receptor-Mediated Inflammatory Responses through Endotoxin Scavenging and Endosomal pH Modulation

Yuting Ji, Liya Sun, Yuan Liu, Yanhui Li, Tongxuan Li, Jiameng Gong, Xiali Liu, Huiqiang Ma, Jingying Wang, Bing Chen, Shan-Yu Fung and Hong Yang**

Supporting Information

Dual functioned hexapeptide-coated lipid-core nanomicelles suppress Toll-like receptor-mediated inflammatory responses through endotoxin scavenging and endosomal pH modulation

Yuting Ji, Liya Sun, Yuan Liu, Yanhui Li,Tongxuan Li, Jiameng Gong, Xiali Liu, Huiqiang Ma, Jingying Wang, Bing Chen, Shan-Yu Fung and Hong Yang

Supporting Information for

Dual functioned hexapeptide-coated lipid-core nanomicelles suppress Toll-like receptor-mediated inflammatory responses through endotoxin scavenging and endosomal pH modulation

Yuting Ji, Liya Sun, Yuan Liu, Yanhui Li,Tongxuan Li, Jiameng Gong, Xiali Liu, Huiqiang Ma, Jingying Wang, Bing Chen, Shan-Yu Fung and Hong Yang

Y. Ji, L. Sun, Y. Liu, Y. Li, J. Gong, H. Ma, H. Yang
The Province and Ministry Co-Sponsored Collaborative Innovation Center for Medical Epigenetics, Department of Pharmacology, School of Basic Medical Sciences, Intensive Care Unit of the Second Hospital, Tianjin Medical University
No. 22 Qixiangtai Road, Heping district, Tianjin 300070, China
E-mail: hongyang@tmu.edu.cn

T. Li, S.Y. Fung
Department of Immunology and Key Laboratory of Immune Microenvironment and Disease (Ministry of Education)
School of Basic Medical Sciences
Tianjin Medical University
No. 22 Qixiangtai Road, Heping district, Tianjin 300070, China
E-mail: shanefung@tmu.edu.cn

X. Liu
Department of Pulmonary and Critical Care Medicine
Shanghai General Hospital
Shanghai Jiao Tong University School of Medicine
No. 650 Xinsongjiang Road, Shanghai 201620, China

J Wang, B. Chen
Intensive Care Unit of the Second Hospital, Tianjin Medical University
No. 22 Qixiangtai Road, Heping district, Tianjin 300070, China

Keywords: lipid nanomicelle, acute lung injury, endotoxin scavenger, toll-like receptor, macrophage, nano-therapy

List of contents for supporting information

1. Supplementary methods

1.1 Materials

1.2. Post-treatment of M-P12 on TLR3-mediated activation of NF-κB/AP-1 and IRF

1.3. Cellular uptake of M-P12 at different temperatures

1.4. DSS-induced ulcerative colitis mouse model

2. Supplementary figures

Figure S1 The physicochemical characterization of M-P12 and Lipo-P12.

Figure S2 The physicochemical characterization of PLGA-P12.

Figure S3 The effects of nano-hybrids on the cell viability of THP-1 reporter cell-derived macrophages.

Figure S4 The effects of M-P12 on the NF-κB/AP-1 and IRF activation in THP-1 reporter cell-derived macrophages.

Figure S5 The physicochemical characterization of M-P13, M-TT and M-SS.

Figure S6 The inhibitory effects of M-P13, M-TT and M-SS on TLR2 signaling pathway.

Figure S7 The inhibitory effects of M-P13 on TLR3 and TLR7/8 signaling pathways.

Figure S8 The inhibitory effects of M-TT on TLR3 and TLR7/8 signaling pathways.

Figure S9 The inhibitory effects of M-SS on TLR3 and TLR7/8 signaling pathways.

Figure S10 P-P12 had no effects on TLR2, TLR3 and TLR7/8 signaling pathways in THP-1 reporter cell-derived macrophages.

Figure S11 The effects of different lipid-core nanomicelles on TLR2, TLR3, TLR5 and TLR7/8 signaling pathways.

Figure S12. Dose-dependent inhibition of M-P12 on TLR4 signaling via scavenging FITC-labelled LPS in THP-1 reporter cell-derived macrophages.

Figure S13 The fluorescence polarization profile of FITC-labelled Poly I:C as a function of M-P12 concentration (phospholipids).

Figure S14 The effects of post-treatment of M-P12 on TLR3 signaling pathway after the removal of Poly I:C stimulation for 2 h.

Figure S15 The quantitative analysis of the uptake of DiD-labeled M-P12 in macrophages at different temperatures.

Figure S16 The effects of M-P13, M-TT and M-SS on the endosomal pH in THP-1 cells-derived macrophages.

Figure S17 The inhibitory effect of $M_{DMPE\text{-}mPEG}$ on the lung inflammation and injury in LPS-induced ALI mice.

Figure S18 The stability of M-P12 and $M_{DSPE\text{-}mPEG}$ over time.

Figure S19 The gating strategy of flow cytometry analysis to identify different immune cells in the lung.

Figure S20 The protective effects of M-P12 on the dextran sulfate sodium (DSS)-induced ulcerative colitis mouse model.

1. Supplementary methods

1.1. Materials

Mouse IL-6 and TNF-α ELISA kits were purchased from InvitroGen (Carlsbad, CA, USA), and mouse KC/CXCL1 ELISA kit was from R&D Systems (Minneapolis, MN, USA). Dextran sulfate sodium (DSS) salt (colitis grade, 36,000 - 50,000 MW) was obtained from MP Biomedicals (Santa Ana, CA, USA).

1.2. Post-treatment of M-P12 on TLR3-mediated activation of NF-κB/AP-1 and IRF

For post-treatment cell model, reporter cell-derived macrophages were first stimulated with Poly I:C (50 μg/mL) for 2 h. Cells were washed three times with PBS to remove Poly I:C, follwed by M-P12 treatment for 24 h. The culture medium was then collected and incubated with QUANTI-Blue solution until the solution color turned into dark blue. The absorption at 655 nm was measured by a microplate reader (TECAN, Mannedorf, Zurich, Switzerland).

1.3. Cellular uptake of M-P12 at different temperatures

To study whether the uptake of M-P12 was energy dependent, THP-1 cells-derived macrophages were treated with DiD-labelled M-P12 for 3.5 h at 4℃ or 37℃; the untreated cells were used as a negative control. Cells were resuspended in PBS for flow cytometry analysis of DiD fluorescence in macrophages on a flow cytometer (BD FACSVerse, BD, San Diego, CA, USA), and the data was processed using FlowJo software (TreeStar, Ashland, OR, USA).

1.4. DSS-induced ulcerative colitis mouse model

Female C57BL/6 mice (8-10 weeks) were randomly divided into Water+PBS group, DSS+PBS group and DSS+M-P12 group. DSS (3%) was prepared in sterilized drinking water and given to the mice for 7 days continuously; mice were then fed with sterilized drinking water for another 2 days. PBS or M-P12 (phospholipids: 7.2 μg/kg) were injected intraperitoneally one day before DSS treatment and every other day during the DSS feeding period (a total of 5 doses).

The colitis severity was scored by evaluating the disease activity through daily observations of the following parameters: weight loss (0 point: ≤ 1%, 1 point: 1-3%, 2 points: 3-6%, 3 points: 6-9%, and 4 points: ≥ 9%), stool consistency (0 point: normal, 1 point: loose, 2 points: very loose, 3 points: diarrhea, and 4 points: no solids) and fecal bleeding (0 point: no

blood, 1 point: observed on the blood test strip, 2 points: visulized trace of blood, 3 points: obvious blood in the stool, 4 points: blood around the stool and the anus). The disease activity index (DAI) was calculated based on the above three parameters to obtain an average value from 0-4. The mice were sacrificed at Day 9, and the colon length was measured; the colon tissues were processed for further analysis.

For histological analyses, half of the distal colon from each mouse was fixed in 4% paraformaldehyde for 24 h, followed by dehydration and paraffin embedding. They were cut into 5-μm thick sections, which were furher processed for hematoxylin and eosin (H&E) staining. The disease severity was evaulated on the basis of a histopathological score that includes 7 parameters: extent of inflammation, inflammatory cell infiltration, extent of crypt damage, crypt abscesses, sub-mucosal edema, loss of goblet cells and reactive epithelial hyperplasia.

A fixed mass of each colon tissue was homogenized in modified RIPA lysis buffer containing phosphotase and protease inhibitors (Beyotime, Shanghai, China) to obtained tissue lysates for cytokine measurements of IL-6, TNF-α and KC/CXCL1 by ELISA kits.

2. Supplementary Figures

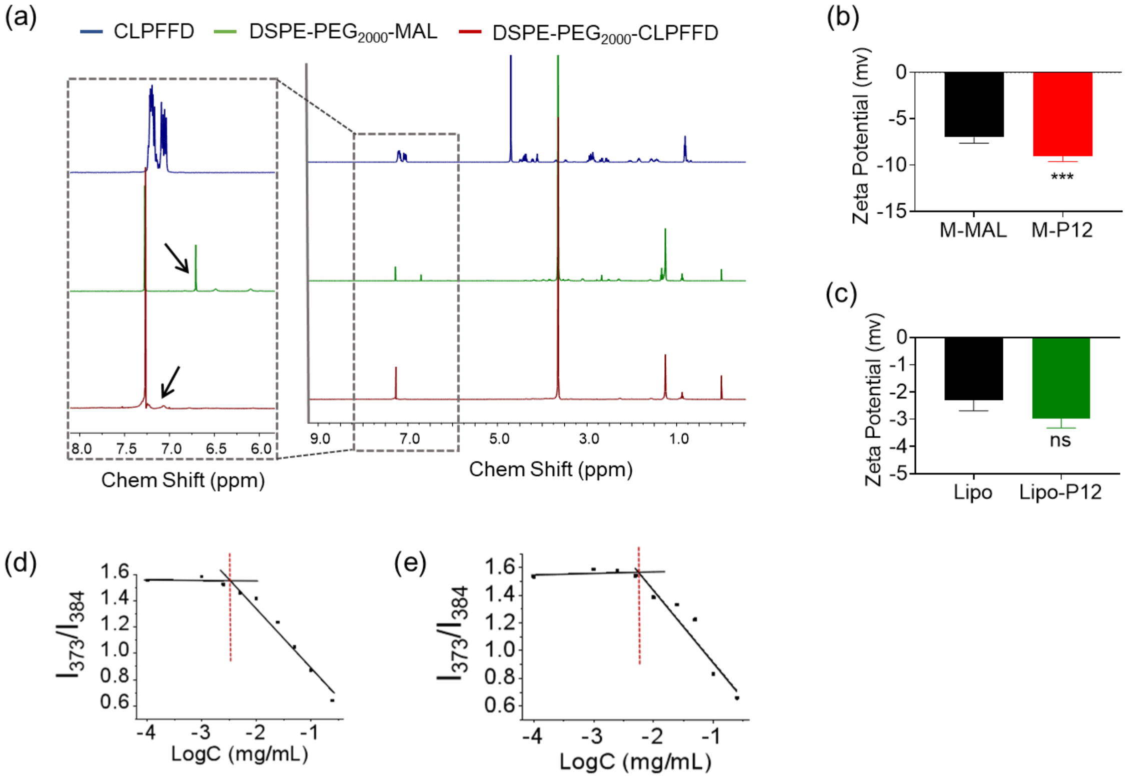

Figure S1. The physicochemical characterization of M-P12 and Lipo-P12. (a) The ^{1}H NMR spectra of the Pep12 (CLPFFD), DSPE-PEG$_{2000}$-MAL and DSPE-PEG$_{2000}$-CLPFFD. The characteristic peak of the maleimide in DSPE-PEG$_{2000}$-MAL and the benzene in DSPE-PEG$_{2000}$-CLPFFD were pointed out by the arrows. (b) Zeta potential of the unmodified (M-Mal) and Pep12 modified (M-P12) DSPE-PEG$_{2000}$-MAL nanomicelles. (c) Zeta potential of unmodified (Lipo) and Pep12 modified (Lipo-P12) liposomes. The CMC measurements of M-P12 (d) and M$_{DSPE\text{-}mPEG}$ (e); the intensity ratios of the first peak to the third peak (I_{373}/I_{384}) of pyrene fluorescence spectrum as a function of the phospholipid concentration; the CMC was obtained at the cross section of the two fitted straight lines. N = 3 for Zeta potential measurement; ns = not significant, ***$p < 0.001$.

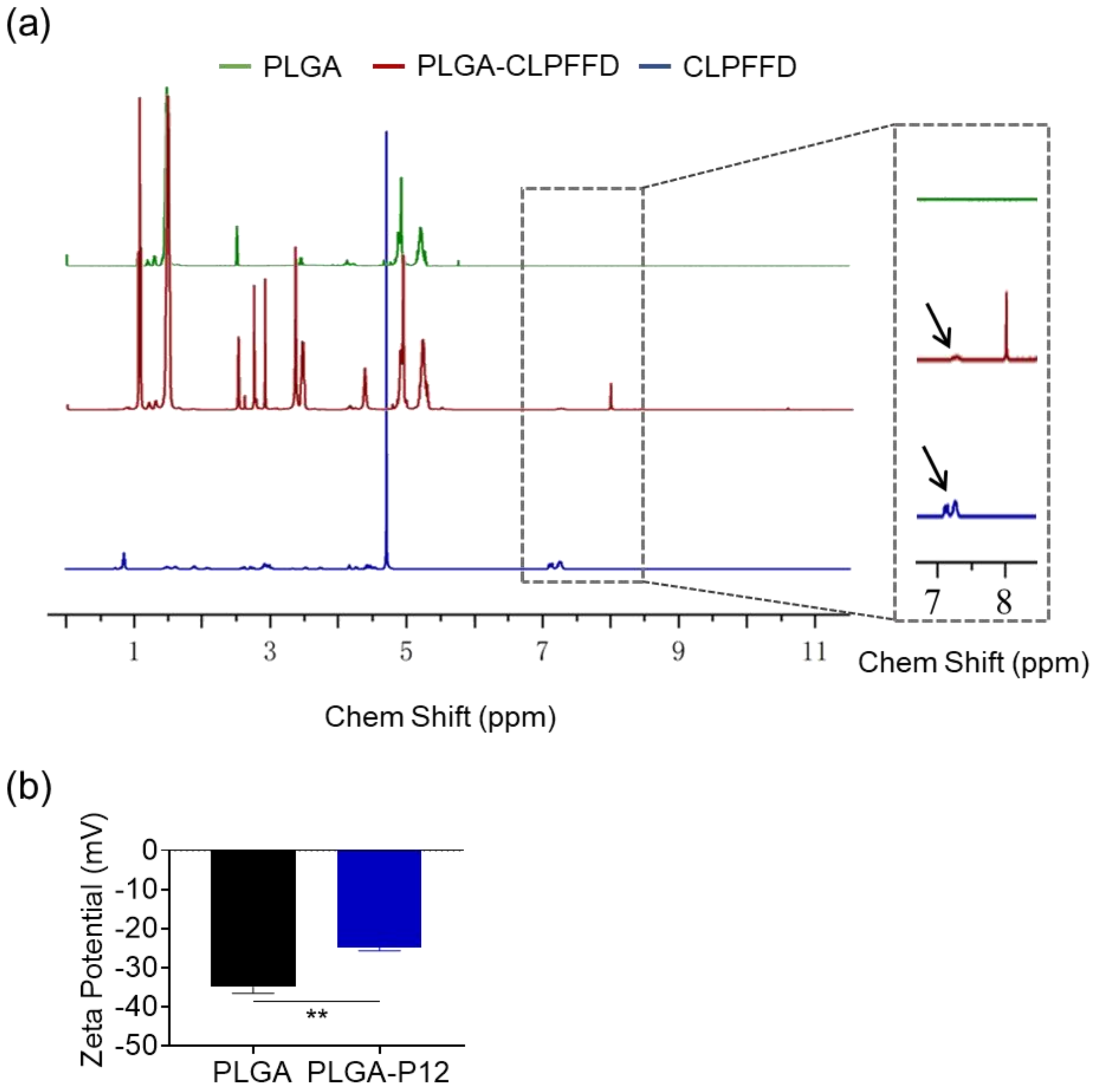

Figure S2. The physicochemical characterization of PLGA-P12. (a) The ^{1}H NMR spectra of PLGA, PLGA-CLPFFD and CLPFFD. The characteristic peak of benzene in PLGA-CLPFFD and CLPFFD were pointed out by the arrows. (b) Zeta potential of unmodified PLGA and PLGA-P12. N = 3; **p < 0.01.

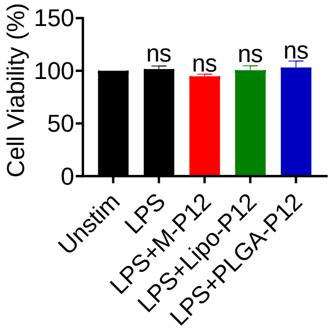

Figure S3. The effects of nano-hybrids on the cell viability of THP-1 reporter cell-derived macrophages. The three nano-hybrids had no effects on the cell viability under LPS stimulation. LPS **=** 10 ng/mL, nano-hybrids (phospholipid/polymer): 0.2 mg/mL; N = 3; ns = not significant vs.Unstim.

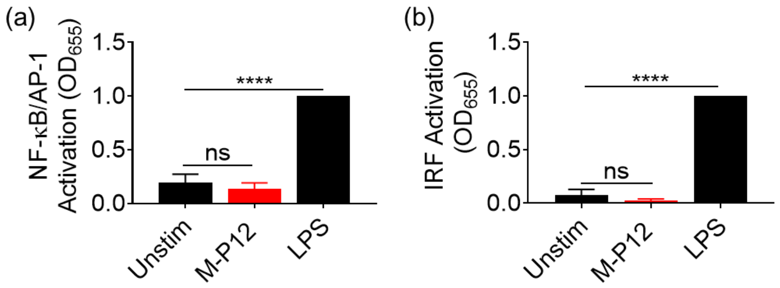

Figure S4. The effects of M-P12 on the NF-κB/AP-1 and IRF activation in THP-1 reporter cell-derived macrophages. M-P12 alone did not induce the activation of NF-κB/AP-1 (a) and IRF (b) in THP-1 reporter cell-derived macrophages; the LPS treatment served as a positive control. LPS = 10 ng/mL, M-P12 (phospholipid): 0.2 mg/mL; N = 3; ns = not significant, ****$p < 0.0001$.

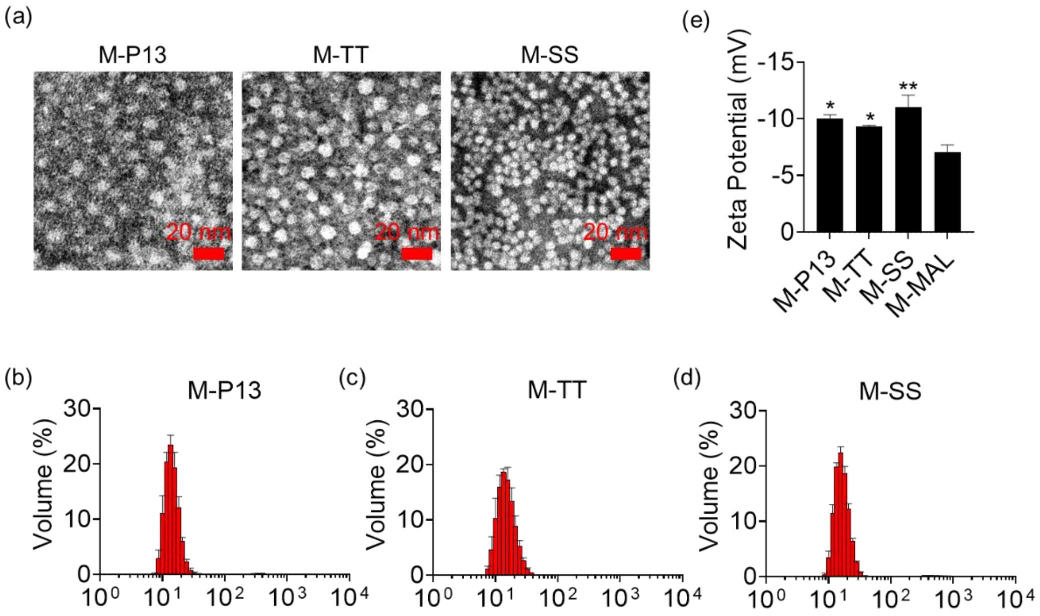

Figure S5. The physicochemical characterization of M-P13, M-TT and M-SS. (a) The TEM images showing the nanostructures of the peptide-conjugated lipid-core nanomicelles; M-P13, M-TT and M-SS displayed a spherical shape with a size of 14.0 ± 2.5, 15.0 ± 2.1, 12.7 ± 1.4 nm, respectively; scale bar = 20 nm. (b-d) The volume-based hydrodynamic size distribution of M-P13 (b), M-TT (c) and M-SS (d) were estimated to be 15.7±1.1, 15.7±0.9 and 17.1±1.1 nm, respectively, by DLS measurements. (e) The Zeta potential of M-P13, M-TT and M-SS in comparison with the unconjugated nanomicelles (M-MAL); N = 3; *p < 0.05, **p < 0.01 vs. M-MAL.

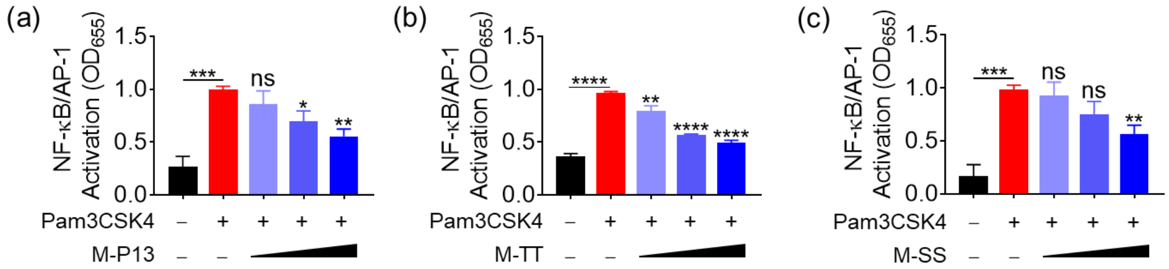

Figure S6. The inhibitory effects of M-P13, M-TT and M-SS on TLR2 signaling pathway. The nanomicelles M-P13 (a), M-TT (b) and M-SS (c) were able to inhibit Pam3CSK4 induced NF-κB/AP-1 activation of TLR2 signaling pathway in a concentration dependent manner. Pam3CSK4 = 10 ng/mL, M-P13/M-TT/M-SS (phospholipids): 0.05, 0.1, 0.2 mg/mL; N = 3; ns = not significant, *p < 0.05, **p < 0.01, ***p < 0.001, ****p < 0.0001 vs. Pam3CSK4 group unless otherwise specified.

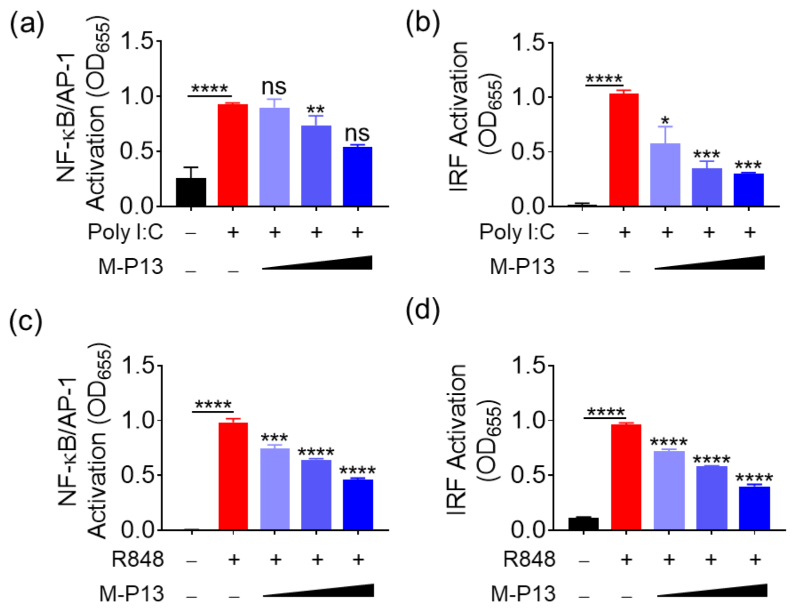

Figure S7. The inhibitory effects of M-P13 on TLR3 and TLR7/8 signaling pathways. The nanomicelles M-P13 could inhibit Poly I:C induced NF-κB/AP-1 (a) and IRF (b) activation of TLR3 signaling pathway and R848 induced NF-κB/AP-1 (c) and IRF (d) activation of TLR7/8 signaling pathway. Poly I:C = 50 μg/mL, R848 = 10 μg/mL, M-P13 (phospholipids): 0.05, 0.1, 0.2 mg/mL; N = 3; ns = not significant, *$p < 0.05$, **$p < 0.01$, ***$p < 0.001$, ****$p < 0.0001$ vs. the positive control groups (Poly I:C or R848) unless otherwise specified.

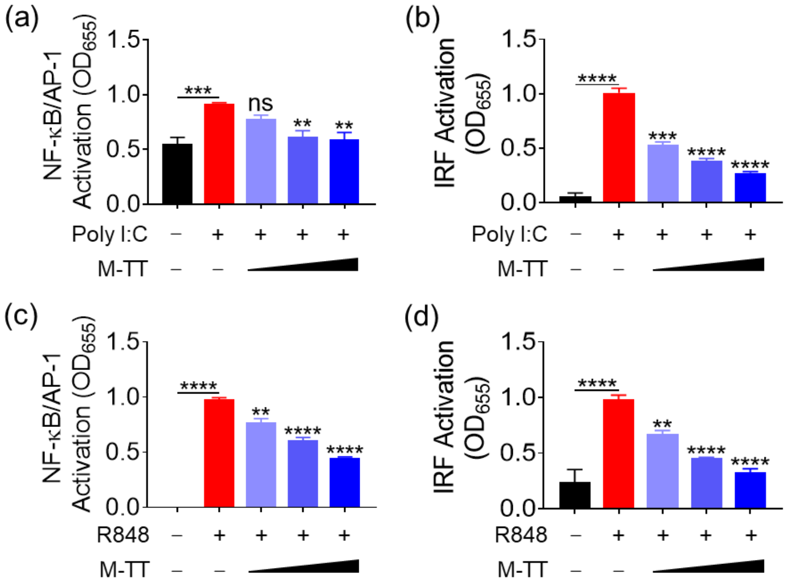

Figure S8. The inhibitory effects of M-TT on TLR3 and TLR7/8 signaling pathways. The nanomicelles M-TT could inhibit Poly I:C induced NF-κB/AP-1 (a) and IRF (b) activation of TLR3 signaling pathway and R848 induced NF-κB/AP-1 (c) and IRF (d) activation of TLR7/8 signaling pathway. Poly I:C = 50 μg/mL, R848 = 10 μg/mL, M-TT (phospholipids): 0.05, 0.1, 0.2 mg/mL; N = 3; ns = not significant, $^{**}p < 0.01$, $^{***}p < 0.001$, $^{****}p < 0.0001$ vs. the positive control groups (Poly I:C or R848) unless otherwise specified.

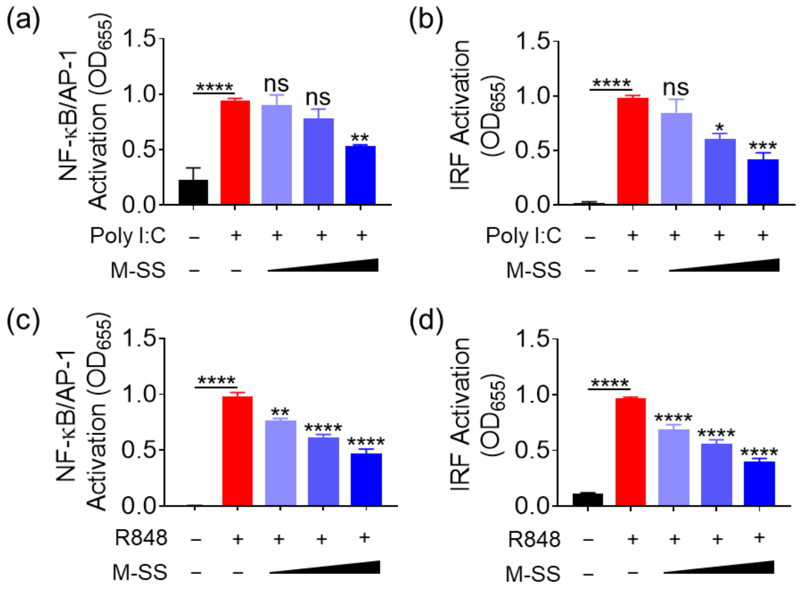

Figure S9. The inhibitory effects of M-SS on TLR3 and TLR7/8 signaling pathways. The nanomicelles M-SS could inhibit Poly I:C induced NF-κB/AP-1 (a) and IRF (b) activation of TLR3 signaling pathway and R848 induced NF-κB/AP-1 (c) and IRF (d) activation of TLR7/8 signaling pathway. Poly I:C = 50 μg/mL, R848 = 10 μg/mL, M-SS (phospholipids): 0.05, 0.1, 0.2 mg/mL; N = 3; ns = not significant, *p < 0.05, **p < 0.01, ***p < 0.001, ****p < 0.0001 vs. the positive control groups (Poly I:C or R848) unless otherwise specified.

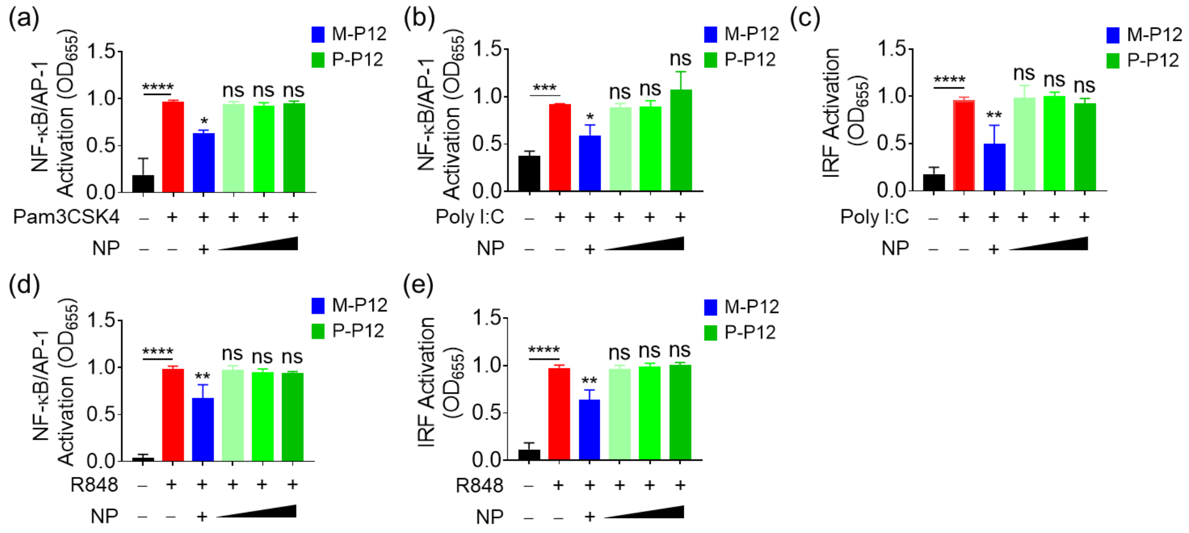

Figure S10. P-P12 had no effects on TLR2, TLR3 and TLR7/8 signaling pathways in THP-1 reporter cell-derived macrophages. The nano-hybrids P-P12 with a polymeric core could not inhibit Pam3CSK4 (TLR2) induced NF-κB/AP-1 activation (a), Poly I:C (TLR3) induced NF-κB/AP-1 (b) and IRF (c) activation and R848 (TLR7/8) induced NF-κB/AP-1 (d) and IRF (e) activation when compared with M-P12 containing a lipid core. Pam3CSK4 = 10 ng/mL, Poly I:C = 50 μg/mL, R848 = 10 μg/mL, M-P12 = 0.2 mg/mL, P-P12: 0.05, 0.1, 0.2 mg/mL; N = 3; ns = not significant, *p < 0.05, **p < 0.01, ***p < 0.001, ****p < 0.0001 vs. the positive control groups (Pam3CSK4, Poly I:C or R848) unless otherwise specified.

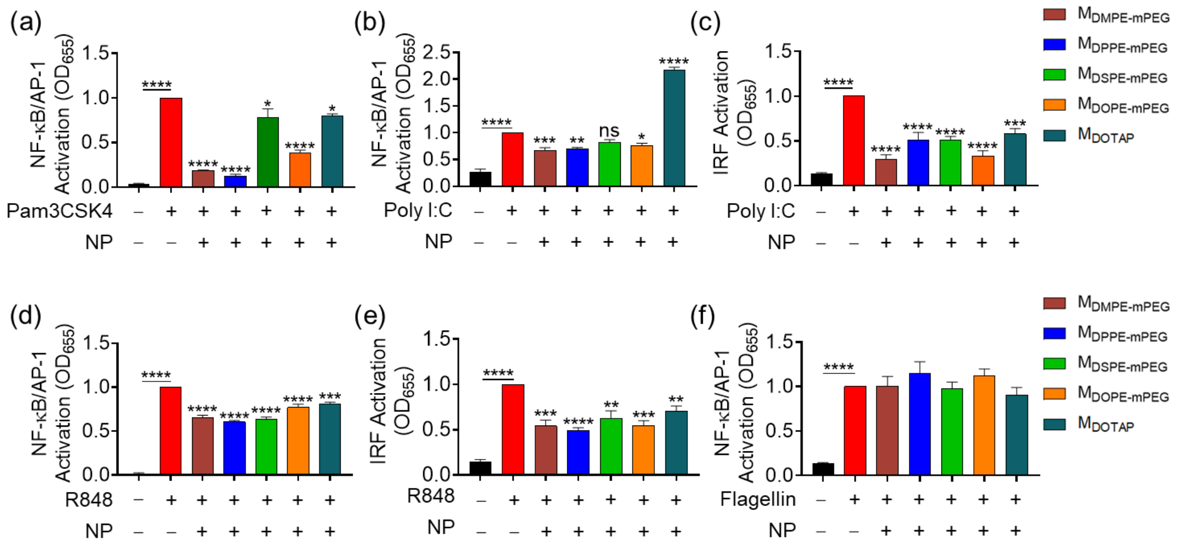

Figure S11. The effects of different lipid-core nanomicelles on TLR2, TLR3, TLR5 and TLR7/8 signaling pathways. The five different lipid-core nanomicelles $M_{DMPE\text{-}mPEG}$, $M_{DPPE\text{-}mPEG}$, $M_{DSPE\text{-}mPEG}$, $M_{DOPE\text{-}mPEG}$ and M_{DOTAP} without peptide modification could inhibit Pam3CSK4 induced NF-κB/AP-1 activation of TLR2 signaling pathway (a), Poly I:C induced NF-κB/AP-1 (b) and IRF (c) activation of TLR3 signaling pathway, and R848 induced NF-κB/AP-1 (d) and IRF (e) activation of TLR7/8 signaling pathway, but not flagellin induced NF-κB/AP-1 activation of TLR5 signaling pathway (f). Pam3CSK4 = 10 ng/mL, Poly I:C = 50 μg/mL, R848 = 10 μg/mL, flagellin = 1 μg/mL, nanomicelles (phospholipids): 0.2 mg/mL; N = 3; ns = not significant, *p < 0.05, **p < 0.01, ***p < 0.001, ****p < 0.0001 vs. the positive control groups (Pam3CSK4, Poly I:C, R848 or flagellin) unless otherwise specified.

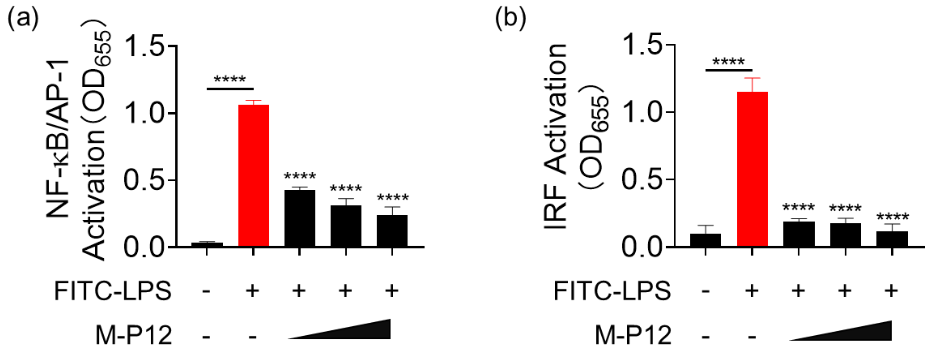

Figure S12. Dose-dependent inhibition of M-P12 on TLR4 signaling via scavenging FITC-labelled LPS in THP-1 reporter cell-derived macrophages. The dose-dependent inhibitory effects of M-P12 on the activation of NF-κB/AP-1 (a) and IRF (b) under FITC-labelled LPS (FITC-LPS) stimulation; FITC-LPS = 10 ng/mL, M-P12 (phospholipids): 0.05, 0.1, 0.2 mg/mL; N = 3, ****$p < 0.0001$ vs. FITC-LPS unless otherwise specified.

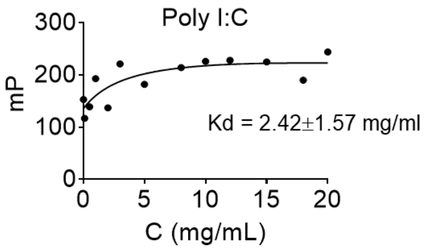

Figure S13. The fluorescence polarization profile of FITC labelled Poly I:C as a function of M-P12 concentration (phospholipids). The non-linear fitting of the profile generated the equilibrium dissociation constant Kd of 2.42 ± 1.57 mg/mL. Poly I:C = 5 μg/mL.

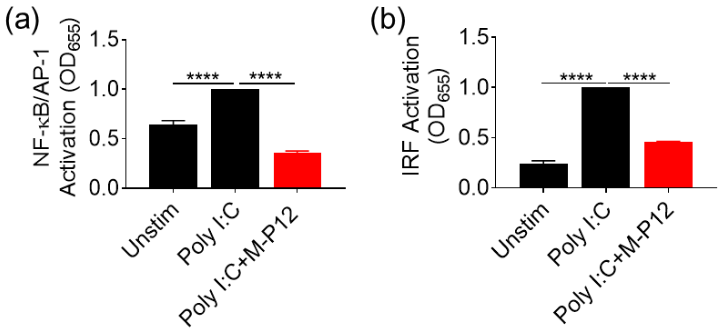

Figure S14. The effects of post-treatment of M-P12 on TLR3 signaling pathway after the removal of Poly I:C stimulation for 2 h. The post-treatment of M-P12 could inhibit Poly I:C induced NF-κB/AP-1 (a) and IRF (b) activation of TLR3 signaling pathways. The reporter cells were stimulated with Poly I:C (50 μg/mL) for 2 h; after the removal of Poly I:C, cells were treated with M-P12 (phospholipids: 0.2 mg/mL) for 24 h. N = 3; ****$p < 0.0001$.

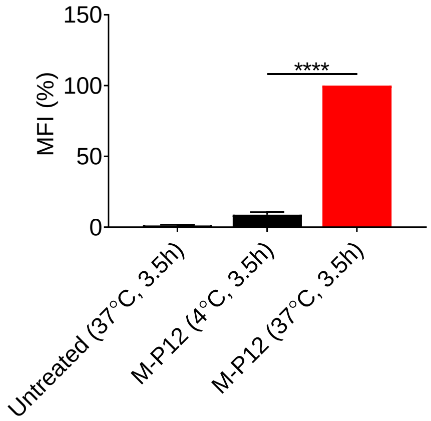

Figure S15. The quantitative analysis of the uptake of DiD-labeled M-P12 in macrophages at different temperatures. The quantitative analysis of DiD fluorescence in the macrophages at 4°C or 37°C. The increase in DiD fluorescence at 37°C indicated an engergy dependent uptake of M-P12 by macrophages. N = 3; ****p < 0.0001.

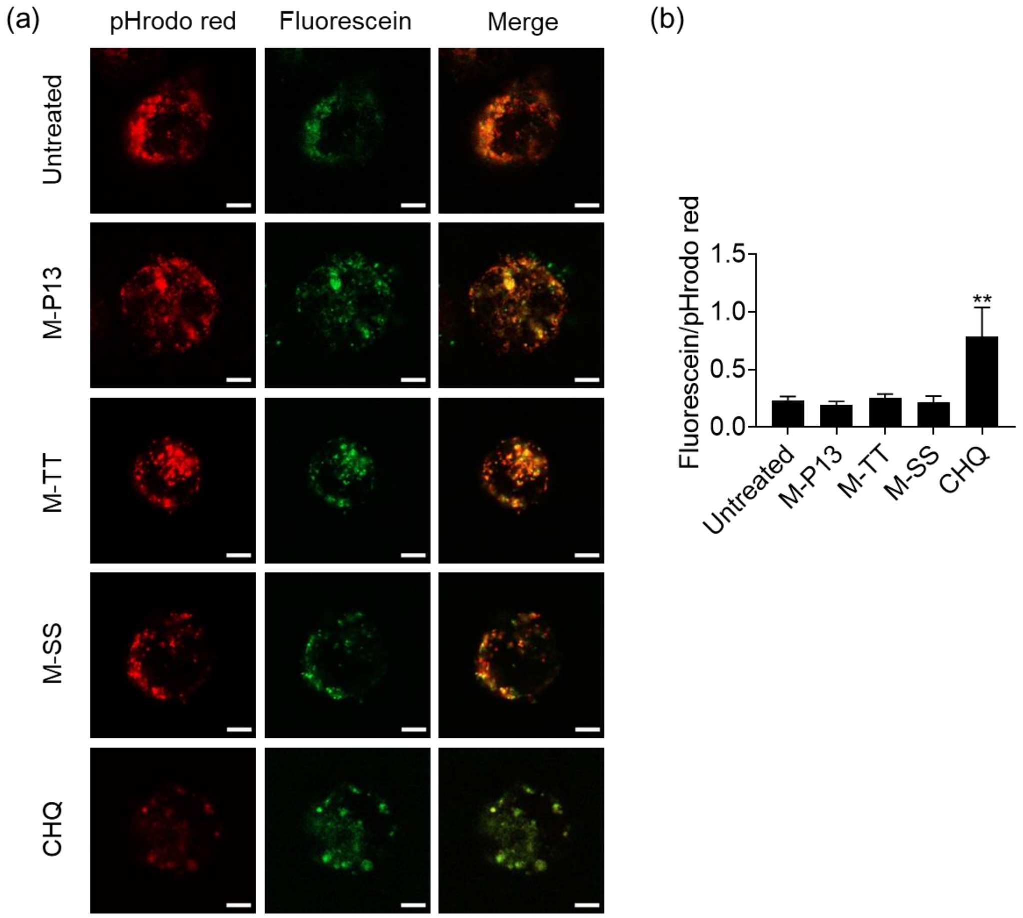

Figure S16. The effects of M-P13, M-TT and M-SS on the endosomal pH in THP-1 cell-derived macrophages. (a) Confocal microscopic images of THP-1 cells-derived macrophages treated with M-P13, M-TT, M-SS or chloroquine (CHQ, 50 μM); the endosomal pH was probed by pHrodo red (red) (10 μg/mL) and fluorescein (green) labeled dextran (20 μg/mL); scale bar = 5 μm. (b) The quantification of the green-to-red ratio of the fluorescence signals; N = 30-36 cells. **$p < 0.01$ vs. untreated group.

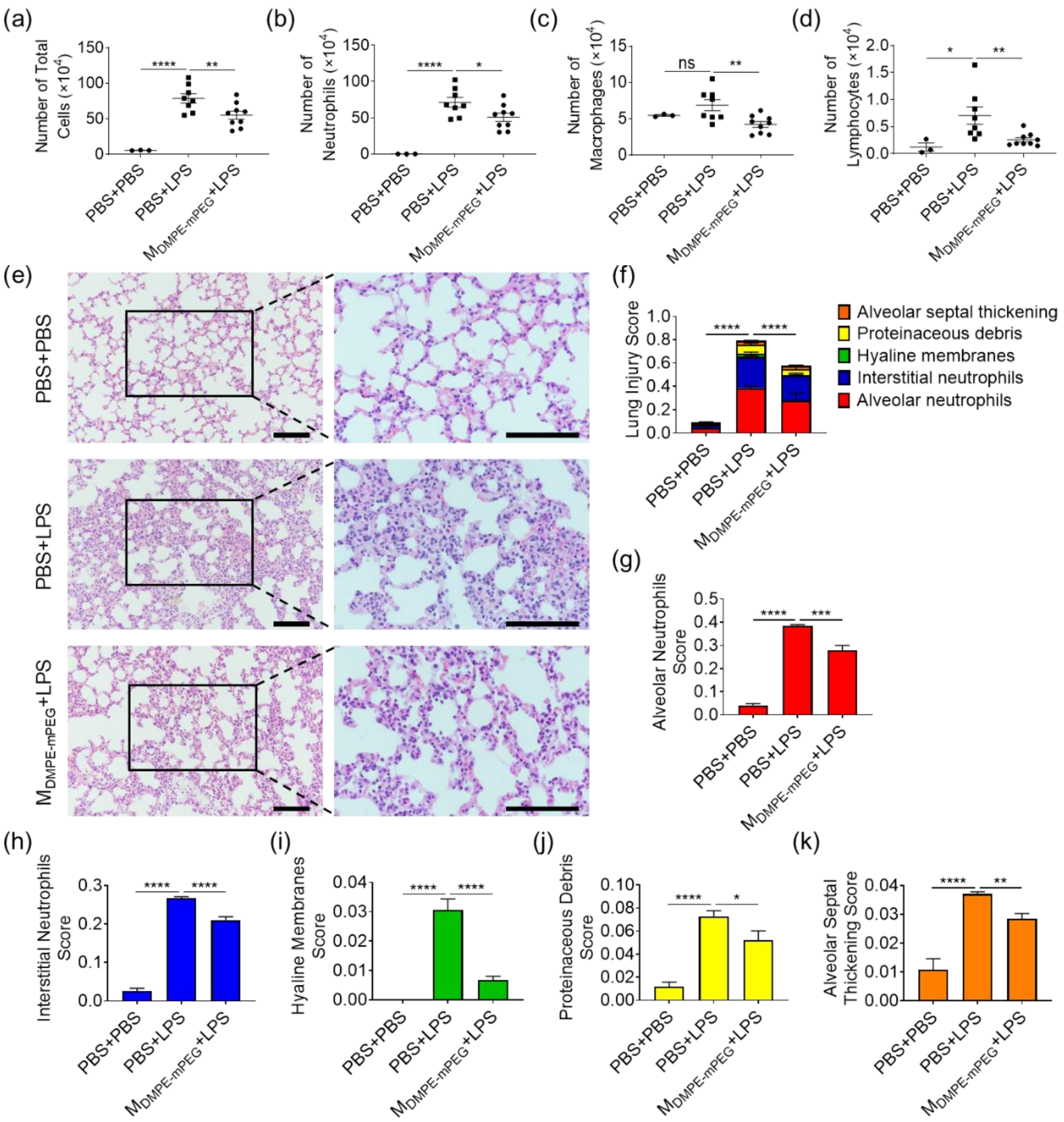

Figure S17. The inhibitory effect of $M_{DMPE\text{-}mPEG}$ on the lung inflammation and injury in LPS-induced ALI mice. (a-d) The BALF was collected for the analysis of the number of total cells (a), neutrophils (b), macrophages (c) and lymphocytes (d) infiltrated in the lung. (e) The images of lung sections stained with H&E; scale bar = 100 μm. (f) The lung injury score based on 5 pathophysiological characteristics: the alveolar neutrophils (g), interstitial neutrophils (h), hyaline membranes (i), proteinaceous debris (j) and alveolar septal thickening (k); N = 3 for PBS+PBS group; N = 5 for other groups. LPS = 10 mg/kg, $M_{DMPE\text{-}mPEG}$ (phospholipids): 0.5 mg/kg; ns = not significant, *$p < 0.05$, **$p < 0.01$, ***$p < 0.001$, ****$p < 0.0001$.

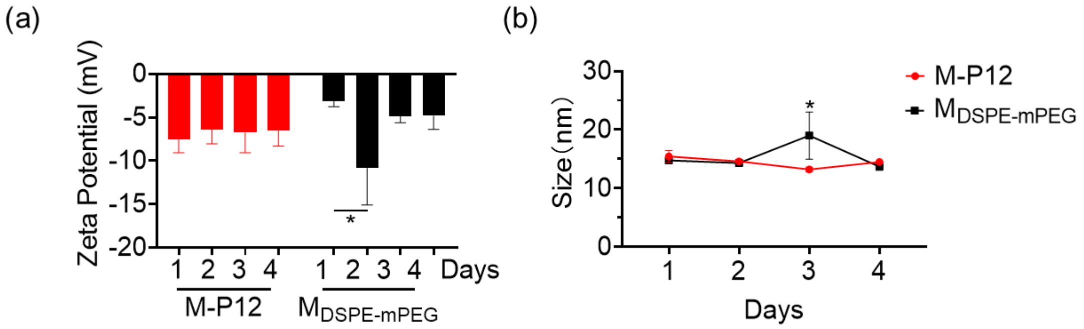

Figure S18. The stability of M-P12 and $M_{DSPE\text{-}mPEG}$ over time. The stability of M-P12 and $M_{DSPE\text{-}mPEG}$ was assessed by the change of Zeta potential (a) and the hydrodynamic size (b) over time. N = 3-4 for (a); N = 2-4 for (b); *$p < 0.05$ vs. 1 Day or $M_{DSPE\text{-}mPEG}$.

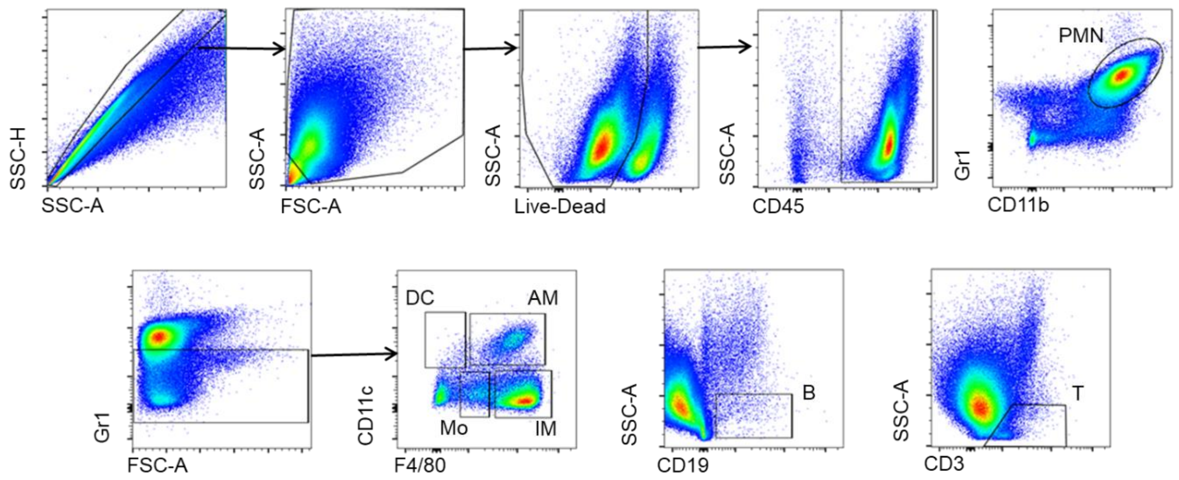

Figure S19. The gating strategy of flow cytometry analysis to identify different immune cells in the lung. Live CD45+ cells were gated to further identify different immune cells based on their unique cell markers: alveolar macrophages (AM, $Gr1^{-}CD11c^{+}F4/80^{+}$), interstitial macrophages (IM, $Gr1^{-}CD11c^{-}F4/80^{+}$), monocyte (Mo, $Gr1^{-}CD11c^{-}F4/80^{low}$), dendritic cells (DC, $Gr1^{-}CD11c^{+}F4/80^{-}$), neutrophils (PMN, $CD11b^{+}Gr1^{+}$), B cells (B, $CD19^{+}$) and T cells (T, $CD3^{+}$).

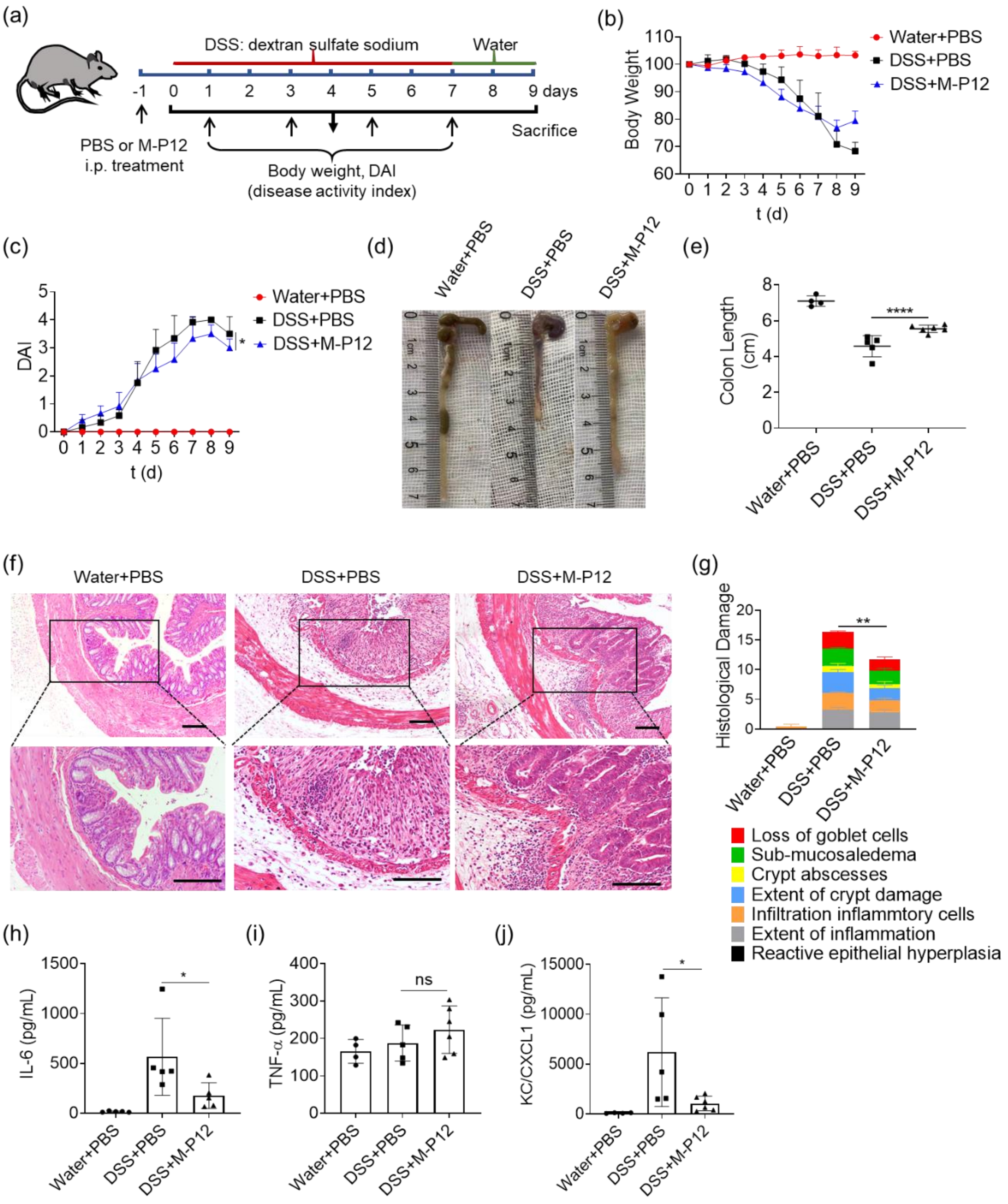

Figure S20. The protective effects of M-P12 on the dextran sulfate sodium (DSS)-induced ulcerative colitis mouse model. (a) The scheme of the DSS-induced colitis model. The disease activity was evaulated by the observation of the change of weight loss (b) and disease activity index (DAI) (c) during the 9-day model period; N = 4-6. (d) The colon length of mice at the end of the model was measured and quantified (e). (f) The histological images of colon sections stained by H&E; scale bar = 50 µm. (g) The disease severity was scored by the histological damage based on 7 parameters: loss of goblet cells, sub-mucosal edema, crypt abscesses, extent of crypt damage, inflammatory cell infiltration, extent of inflammation, and

reactive epithelial hyperplasia; N = 5. The cytokine levels of IL-6 (h), TNF-α (i) and KC/CXCL1 (j) in the colon. DSS: 3% in the drinking water, M-P12 (phospholipids): 7.2 μg/kg; ns = not significant, *$p < 0.05$, **$p < 0.01$, ****$p < 0.0001$.
